# Breaking the activity-stability limit in acidic oxygen evolution reaction with dual-iridium active sites

**DOI:** 10.1126/sciadv.aee7510

**Published:** 2026-04-15

**Authors:** Lei Tang, Yunchuan Tu, Mingyue Zhang, Yan Yan, Shibo Xi, Yan Liu, Yijiang Liu, Javier Pérez-Ramírez, Zhiqun Lin

**Affiliations:** ^1^ Department of Chemical and Biomolecular Engineering, National University of Singapore, 4 Engineering Drive 4, Singapore 117585, Singapore.; ^2^ Institute of Sustainability for Chemicals, Energy and Environment (ISCE^2^), Agency for Science, Technology and Research (A*STAR), 1 Pesek Road, Jurong Island, Singapore 627833, Republic of Singapore.; ^3^ School of Chemistry and Chemical Engineering, Chongqing University, Chongqing 400044, PR China.; ^4^ School of Chemistry and Chemical Engineering, Anhui University of Technology, Ma’anshan, Anhui 243002, China.; ^5^ College of Chemistry and Key Laboratory of Environmentally Friendly Chemistry and Application of Ministry of Education, Xiangtan University, Xiangtan, Hunan Province 411105, China.; ^6^ Institute for Chemical and Bioengineering, Department of Chemistry and Applied Biosciences, ETH Zurich, Vladimir-Prelog-Weg 1, Zurich 8093, Switzerland.; ^7^ Department of Chemistry and Nanoscience, College of Natural Sciences, Ewha Womans University, Seoul 03760, Republic of Korea.

## Abstract

Meeting terawatt-scale global energy demands requires overcoming the activity-stability trade-off of iridium-based catalysts, the only industrially viable option for acidic water oxidation. Herein, we report a lattice-confinement strategy that creates patches of fully exposed Ir atoms, simultaneously enhancing oxygen evolution reaction (OER) activity and stability in acidic media. In situ vibrational and mass spectroscopy identify peroxo intermediates bridging adjacent Ir atoms, supporting an OER mechanism based on direct O─O radical coupling. Density functional theory reveals synergistic interactions between neighboring Ir atoms that enable O_2_ evolution through a low-energy pathway. These abundant dual active sites achieve an ultralow overpotential of 198 millivolts at 10 milliamperes per square centimeter and a two-order-of-magnitude higher mass activity than commercial IrO_2_. Stability calculations further show that lattice confinement suppresses Ir dissolution, enabling durable operation beyond 2 months in acid. This work establishes lattice confinement as an effective route to high-performance catalysts for practical water electrolysis.

## Teaser

Lattice-confined Ir patches boost acidic OER by preventing dissolution and enabling dual active sites for activity and stability.

## INTRODUCTION

Proton exchange membrane water electrolysis (PEMWE) in acidic electrolytes is considered ideal for hydrogen energy production due to its high efficiency and ability to achieve large current density over conventional alkaline water electrolysis (*1*–*4*). However, the sluggish reaction kinetics of the oxygen evolution reaction (OER) at the PEMWE anode, in conjunction with the poor durability of traditional OER catalysts in acidic environments, greatly hinder the large-scale development and commercial application of PEMWE (*5*–*9*). The past decades have witnessed rapid advances in iridium (Ir)–based catalysts, which remain the only industrially viable option for acidic OER (*10*–*13*). Although they offer superior stability to ruthenium- and cobalt-based catalysts, they manifest suboptimal activity and face a notable trade-off between activity and stability under acidic conditions (*11*, *14*–*17*). Furthermore, the scarcity of Ir necessitates substantial improvements in its activity and stability to maximize Ir utilization and advance progress toward sustainable development goals.

**Fig. 1.**
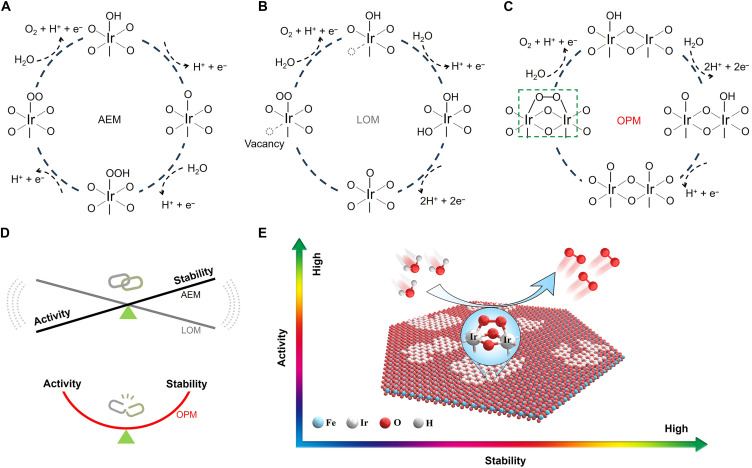
Tailoring activity and stability through modulation of the OER mechanism. (**A** to **C**) Schematic illustrations of the (A) AEM, (B) LOM, and (C) OPM, respectively, toward acidic OER. (**D**) Schematic comparison of the activity-stability trade-off in acidic OER under AEM and LOM, and the alleviation of this trade-off via OPM; the interlocked clips (top) symbolize the conventional constraint between activity and stability, whereas the separated clips (bottom) represent its release. (**E**) Schematic illustration of dual-Ir active sites simultaneously enhancing OER activity and stability.

From a mechanistic viewpoint, the OER on catalyst surfaces is commonly understood to follow the conventional adsorbate evolution mechanism (AEM) (*18*–*20*), involving the adsorption and subsequent desorption of various oxygenated intermediates (such as *OH, *O, and *OOH; Fig. 1A) on a single-metal site. When the AEM-based OER process occurs on the metal oxide surface, the binding energies of intermediates demonstrate a linear relationship, in accordance with the proportional correlation (ΔE_*OOH_ − ΔE_*OH_ = 3.2 eV) (*21*, *22*). The binding energy of each intermediate cannot be independently adjusted, resulting in the necessity of a high overpotential to drive the reaction, with a theoretical limit of ~370 mV (*23*, *24*). Recent studies indicate that the overpotential of catalysts can be substantially reduced when the OER process on the catalyst surface follows the lattice oxygen–mediated mechanism (LOM) (*25*–*28*). This proposed mechanism, in which lattice oxygen directly participates in the reaction, offers an alternative OER pathway with activity surpassing the theoretical limit (Fig. 1B). However, the OER process relying on the LOM mechanism induces a notable presence of oxygen vacancies on the catalyst surface, leading to the detachment of metal species and consequently accelerating the rapid degradation of electrocatalyst (*29*–*31*). In this context, a unique oxide path mechanism (OPM) has recently been proposed (*32*, *33*), using dual-metal sites as active catalytic sites. In the OPM, two neighboring metal sites are strategically positioned to independently generate two *O species, enabling their coupling and subsequent release of oxygen molecules (Fig. 1C). Such direct O─O coupling in the oxygen evolution bypasses the formation of *OOH intermediates, allowing the OPM-based OER process to overcome the linear relationship constraint seen in traditional AEM. This reduces the overpotential required for oxygen evolution, thereby enhancing OER activity. Furthermore, the stability of the catalyst is retained as lattice oxygen on the catalyst’s surface does not directly engage in the OER process within the OPM pathway. Thus, designing electrocatalysts based on the OPM mechanism is envisaged as a promising strategy to overcome the trade-off between activity and stability (Fig. 1D). Although this advancement marks a notable step in developing highly active and acid-stable OER catalysts, the precise interplay among the OPM (i.e., dual-metal site) mechanism, OER activity, and OER stability remains not fully understood.

Herein, we develop a lattice confinement strategy to construct patches of fully exposed iridium (Ir) atoms with abundant dual-Ir sites that promote the OPM mechanism, demonstrating concurrent superior activity and stability under acidic conditions (Fig. 1E). Notably, this lattice-confined Ir atom patch requires only 198 mV of overpotential to achieve a current density of 10 mA cm^−2^. Moreover, this fully used Ir atom patch manifests a two-order-of-magnitude improvement in mass activity over commercial IrO_2_, potentially reducing Ir consumption in large-scale applications. Further scrutiny via in situ infrared (IR) and mass spectrometry studies reveals the bridging of peroxo species on the dual-Ir sites, signifying that the OER on the Ir atom patch proceed via the dual-active site mechanism without the generation of *OOH intermediate. Furthermore, density functional theory (DFT) calculations suggest that concerted interaction between two adjacent Ir atoms facilitates O_2_ evolution through a low-energy reaction pathway featured by direct O─O radical coupling. Stability calculations further insinuate that the Ir atom patch thermodynamically prevents dissolution, leading to a lifetime exceeding thousands of hours. Our findings underscore the robustness of the lattice confinement strategy in creating OER catalysts with both high activity and stability in acidic water oxidation, accentuating the crucial role of dual active sites in catalyst design.

## RESULTS

### Synthesis and characterization of Ir atom patches

**Fig. 2.**
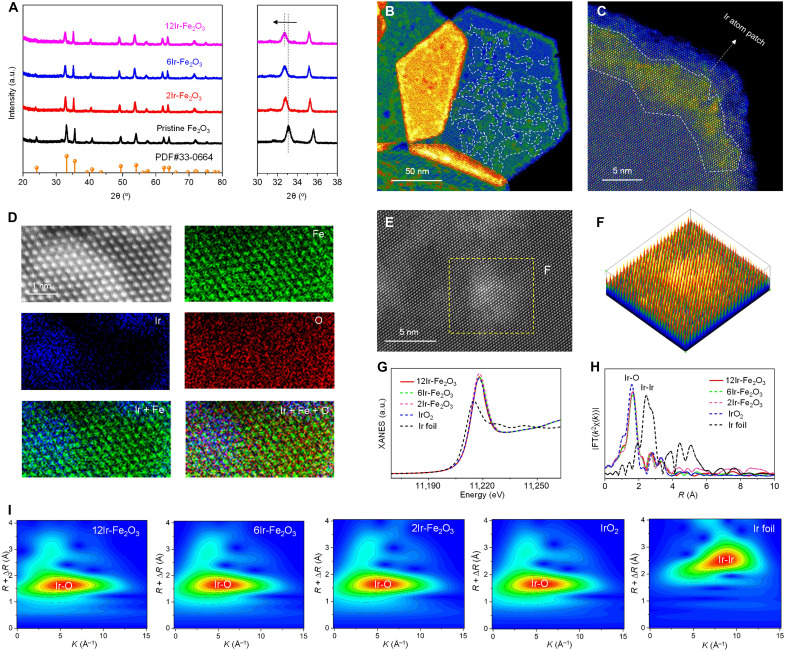
Characterizations of the Ir atom patch catalyst. (**A**) XRD patterns of Fe_2_O_3_ and Ir-Fe_2_O_3_ catalysts. The magnified pattern on the right displays the two most intense diffraction peaks. (**B**) HAADF-STEM image of 12Ir-Fe_2_O_3_, revealing numerous island-like patches (indicated by white dashed lines) distributed across the surface of the Fe_2_O_3_ nanosheet. (**C**) Atomic-resolution HAADF-STEM image of 12Ir-Fe_2_O_3_. (**D**) Atomic-resolution elemental mapping of 12Ir-Fe_2_O_3_. (**E**) High-magnification HAADF-STEM image of 12Ir-Fe_2_O_3_. (**F**) 3D pseudocolor surface plot (boxed area of E). (**G**) Normalized XANES spectra at the Ir L_3_ edge of 12Ir-Fe_2_O_3_, 6Ir-Fe_2_O_3_, 2Ir-Fe_2_O_3_, Ir foil, and IrO_2_. (**H**) FT-EXAFS spectra for the Ir L_3_ edge of 12Ir-Fe_2_O_3_, 6Ir-Fe_2_O_3_, 2Ir-Fe_2_O_3_, Ir foil, and IrO_2_. (**I**) Wavelet transform of Ir L_3_ edge data for 12Ir-Fe_2_O_3_, 6Ir-Fe_2_O_3_, 2Ir-Fe_2_O_3_, Ir foil, and IrO_2_. a.u., arbitrary units.

Although Ir offers excellent stability in acidic environments, its scarcity hinders large-scale application; anchoring it on the surface of a low-cost, abundant support not only minimizes Ir usage but also enables it to shield the underlying carrier. α-Fe_2_O_3_ nanosheets, used as support materials, were synthesized via the hydrothermal approach as previously reported (*34*). The nanosheet-like structure was substantiated by scanning electron microscopy (SEM) and transmission electron microscopy (TEM) (figs. S1 and S2), whereas x-ray diffraction (XRD) confirmed the predominant exposure of {001} facets in the as-prepared α-Fe_2_O_3_ nanosheets (Fig. 2A). The α-Fe_2_O_3_(001) surface features numerous threefold hollow sites created by the arrangement of three surface lattice oxygen atoms, as illustrated in fig. S3. The Fe-vacancy sites have demonstrated efficacy in immobilizing heteroatoms with ionic radii of ~0.6 Å, such as Mo^5+/6+^ and W^5+/6+^ (*34*, *35*). Given that the ionic radius of Ir^4+^ is also ~0.6 Å, we hypothesize that Ir^4+^ can similarly be adsorbed and incorporated into the surface lattice of α-Fe_2_O_3_ nanosheets. To verify this hypothesis, we immersed the as-prepared α-Fe_2_O_3_ nanosheets into an aqueous solution of hexachloroiridate (H_2_IrCl_6_) for adsorption. Following a period of mixing and stirring, centrifugation was performed, and it was observed that the characteristic color of Ir^4+^ was no longer present in the supernatant (fig. S4). This experimental result provides direct evidence that Ir^4+^ adsorbs on the surface of α-Fe_2_O_3_ nanosheets. The acidic nature of the chloroiridic acid precursor induces partial etching of the Fe_2_O_3_ surface, generating additional Fe vacancies that facilitate the incorporation of Ir atoms into the lattice during subsequent calcination. Furthermore, the loading quantity of Ir on the α-Fe_2_O_3_ nanosheets can be modulated by adjusting the concentration of the H_2_IrCl_6_ precursor. Accordingly. three samples, designated as Ir-Fe_2_O_3_, were synthesized with Ir mass loadings of 1.8, 6.3, and 12.1 wt %, as determined by inductively coupled plasma optical emission spectroscopy (ICP-OES). These samples are labeled as *x*Ir-Fe_2_O_3_ (*x* = 2, 6, and 12), where the variable *x* represents the weight percentage of Ir. The crystal structures of the pristine α-Fe_2_O_3_ nanosheets, along with those of 2Ir-Fe_2_O_3_, 6Ir-Fe_2_O_3_, and 12Ir-Fe_2_O_3_, were analyzed using XRD, as shown in Fig. 2A. In all cases, the diffraction patterns correspond to a single phase of Fe_2_O_3_ (Joint Committee on Powder Diffraction Standards number 33-0664), confirming that the incorporation of Ir did not alter the crystal structure. Even with a 12% Ir loading, no additional impurity phases were detected by XRD. This result suggests that Ir is predominantly incorporated into the outer surface of the Fe_2_O_3_ nanosheets, rather than within the bulk phase (*36*). Moreover, because of the larger atomic radius of Ir than Fe, substituting Fe with Ir leads to a shift of the peak in the pristine Fe_2_O_3_ nanosheets toward lower angles. Because XRD reflects the bulk-averaged lattice structure, the observed diffraction peak shift indicates that Fe→Ir substitution extends beyond the outermost surface and partially penetrates into the near-surface and shallow bulk lattice. SEM and TEM characterization further substantiated that the introduction of Ir did not affect the morphology of the nanosheets (figs. S5 and S6). These findings preliminarily indicate that Ir can adsorb onto the surface of Fe_2_O_3_ nanosheets and integrate into their crystal lattice.

The aberration-corrected high-angle annular dark-field scanning TEM (HAADF-STEM) technique was used to characterize the precise localization of the introduced Ir element on the surface of 12Ir-Fe_2_O_3_ nanosheets. It reveals the presence of numerous white flakes resembling isolated islands distributed across the nanosheets (fig. S7). To facilitate the identification of the “nanoislands” formed on the nanosheets, we applied colorization to the HAADF-STEM images. As depicted in Fig. 2B, the white dotted lines delineate numerous green flake-shaped “nanoislands.” Atomic-resolution HAADF-STEM images, when viewed at increased magnification, reveal that the observed sheetlike “nanoislands” consist of overlayer formed by individual atoms (Fig. 2C and fig. S8). Because of the significantly larger atomic radius of Ir compared to Fe, the distinctly bright atomic overlayer can be attributed to the Ir atomic arrangements (*37*). This conclusion was further substantiated by atomically resolved elemental mapping. As shown in Fig. 2D and fig. S9, the Fe-resolved mapping suggests a uniform dispersion of Fe atoms throughout the designated region, whereas the Ir-resolved mapping indicates exclusive distribution of Ir atoms within the corresponding high-brightness regions in the HAADF image. The overlaid maps unveil a systematically arranged atomic configuration and a consistent geometric profile, closely mirroring that of the corresponding HAADF image. The presence of Ir within the corresponding highly illuminated region in HAADF image provides experimental evidence that these atomic aggregates consist of patches of Ir atoms. The discernible convexity of the nanosheet surface is evident in the corresponding three-dimensional (3D) pseudocolor representation (Fig. 2, E and F), suggesting the presence of ordered patch-like substances distributed across the Fe_2_O_3_ surface. The atomic-resolution TEM characterization indicates that the high Ir content sample (12Ir-Fe_2_O_3_) predominantly forms an ordered atomic patch of Ir on the surface lattice of Fe_2_O_3_ nanosheets. In contrast, the low Ir content sample (2Ir-Fe_2_O_3_) is primarily present as single atoms on the Fe_2_O_3_ nanosheet surface (see fig. S10).

The chemical state and coordination structure of the Ir species were investigated using x-ray absorption fine structure (XAFS) spectroscopy and x-ray photoelectron spectroscopy (XPS). As depicted in Fig. 2G, x-ray absorption near-edge structure (XANES) spectra at the L_3_ edge for samples with varying Ir contents exhibit features akin to IrO_2_ and diverge from the profiles observed for Ir foil, signifying the consistent valence state of the introduced Ir element with that of Ir in IrO_2_. This outcome aligns with the XPS findings (fig. S11). Similar to the +4 valence characteristic XPS peak observed in IrO_2_, the double shoulder peaks in the XPS spectra of 2Ir-Fe_2_O_3_, 6Ir-Fe_2_O_3_, and 12Ir-Fe_2_O_3_ can be deconvoluted into four peaks centered at 62.1, 63.1, 65.0, and 66.0 eV (*38*). The local coordination environment of the Ir atom was further scrutinized using extended x-ray absorption fine structure (EXAFS). Figure 2H displays the Fourier transformation (FT) of the EXAFS (FT-EXAFS) spectra for the Ir-Fe_2_O_3_ catalysts, along with IrO_2_ and Ir foil for comparison. The FT-EXAFS profiles of the Ir-Fe_2_O_3_ catalysts exhibit a distinct peak at ~1.6 Å, indicative of the first Ir─O coordination shell. In addition, the peak at 2.8 Å can be attributed to the second coordination shell involving Ir─Fe and/or Ir─Ir paths. To enhance the discernment of coordination configurations around Ir atoms in the Ir-Fe_2_O_3_ catalyst, we conducted wavelet transform analysis on the EXAFS data (Fig. 2I). Notably, there is no intensity maximum at ~8 Å^−1^ in Ir-Fe_2_O_3_; instead, a singular intensity maximum at around 5 Å^−1^ is observed, further proving that Ir is present in the oxide form. The least-squares FT-EXAFS fitting for 12Ir-Fe_2_O_3_ gives an Ir─O coordination number of 5.9, with an average bond length of 2.01 Å in the first shell (figs. S12 and S13 and table S1). The fitting of 2Ir-Fe_2_O_3_ and 6Ir-Fe_2_O_3_ indicates the structural parameters in the first shell analogous to those of 12Ir-Fe_2_O_3_. Nevertheless, in the second shell, a change in the coordination number is observed, where the dominance of Ir─Ir coordination is gradually replaced by Ir─Fe coordination, particularly at lower Ir mass loadings. As the Ir loading increases, the number and lateral size of Ir atom patches increase, such that the Ir─Ir coordination contribution in the second shell becomes dominant for the highly loaded 12Ir-Fe_2_O_3_ catalyst. The aforementioned findings corroborate the results from atomic-resolution TEM, where the high Ir content (12Ir-Fe_2_O_3_) mainly occupies Fe vacancies within the surface lattice of Fe_2_O_3_ to form atomic patches in the form of oxides. In contrast, the lower Ir content (2Ir-Fe_2_O_3_) primarily exists as single atoms within the Fe_2_O_3_ surface lattice. Furthermore, this strategy for preparing lattice-confined atomic patches via pretreatment of the support with chloroiridium acid should be extendable to other metal oxide supports and noble metals. The vacancy structure and lattice symmetry of the support, together with the ionic radius of the metal precursor, are likely to be the key factors governing its general applicability.

### Assessment of catalyst activity

**Fig. 3.**
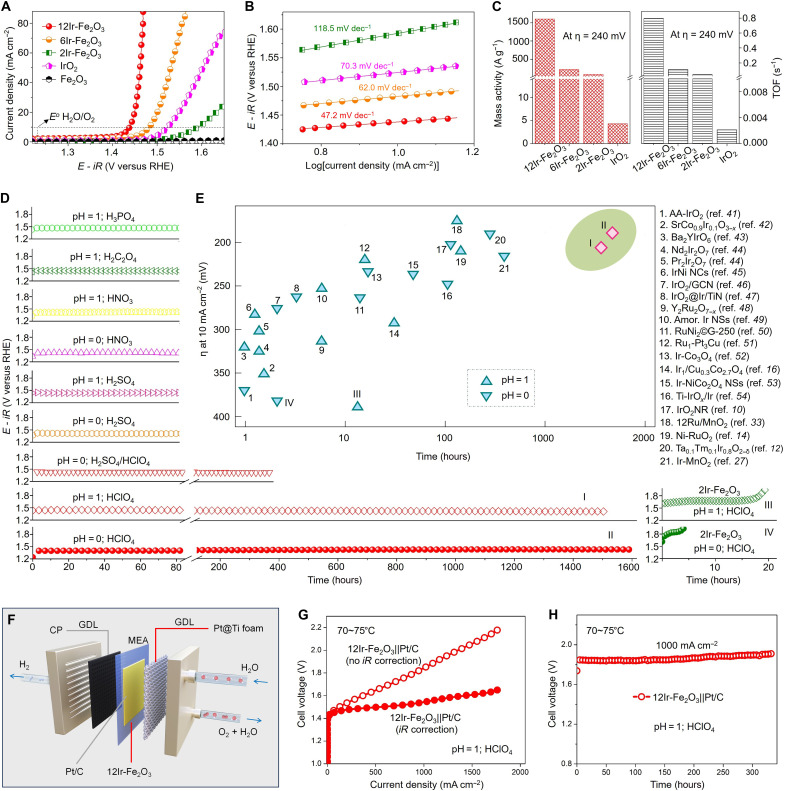
Superior activity and stability of 12Ir-Fe_2_O_3_ among the tested catalysts. (**A**) The OER polarization curves of *x*Ir-Fe_2_O_3_ (*x* = 2, 6, and 12), Fe_2_O_3_, and IrO_2_ in 1 M HClO_4_. (**B**) Tafel slope derived from (A). (**C**) Mass activities and TOF of different samples, calculated according to the Ir loadings. (**D**) Time dependence of the electrochemical potential necessary to perform OER at 10 mA cm^−2^ in H_3_PO_4_ (pH = 1), H_2_C_2_O_4_ (pH = 1), HNO_3_ (pH = 1), HNO_3_ (pH = 0), H_2_SO_4_ (pH = 1), H_2_SO_4_ (pH = 0), H_2_SO_4_/HClO_4_ (pH = 0), HClO_4_ (pH = 1), and HClO_4_ (pH = 0), respectively. The stabilities of 2Ir-Fe_2_O_3_ in HClO_4_ (pH = 1) and HClO_4_ (pH = 0) are shown for comparison. (**E**) Comparison of the stability of 12Ir-Fe_2_O_3_ with 2Ir-Fe_2_O_3_ and other noble metal–based OER catalysts reported in the literature. The measurements were performed under acidic conditions. Data points labeled with Roman numerals (I to IV) correspond to curves I to IV in (D). (**F**) Schematic diagram of a proton exchange membrane water electrolyzer (MEA, membrane electrode assembly; CP, carbon paper; Pt@Ti foam, Pt-coated titanium foam). (**G**) Polarization curves of the PEM electrolyzers using 12Ir-Fe_2_O_3_ as anode catalysts with and without *iR* correction. (**H**) Chronopotentiometry curve of the PEM electrolyzer using 12Ir-Fe_2_O_3_ catalyst operated at 1000 mA cm^−2^.

In accordance with established OER standard procedures, we first evaluated the OER activities of the 2Ir-Fe_2_O_3_, 6Ir-Fe_2_O_3_, and 12Ir-Fe_2_O_3_ catalysts, along with relevant control samples, in a 1 M HClO_4_ electrolyte (pH = 0), using a conventional three-electrode configuration. The control samples are pristine Fe_2_O_3_ nanosheets and commercially available IrO_2_ nanoparticles. Linear sweep voltammetry (LSV) analysis revealed negligible OER activity in pristine Fe_2_O_3_ nanosheets (Fig. 3A and fig. S14). The overpotential of commercial IrO_2_ at a current density of 10 mA cm^−2^ is 295 mV, consistent with previously reports (*39*, *40*). Among the tested catalysts, the 12Ir-Fe_2_O_3_ catalyst exhibits superior catalytic activity, necessitating only an overpotential of 198 mV to attain a current density of 10 mA cm^−2^ (with *iR* correction). This overpotential is lower than that of the 6Ir-Fe_2_O_3_ catalyst (253 mV) and the 2Ir-Fe_2_O_3_ catalyst (360 mV). The observed overpotential disparity was further accentuated with increasing current densities, owing to the markedly decreased Tafel slope of 12Ir-Fe_2_O_3_ (47.2 mV dec^−1^), compared to the Tafel slopes of 6Ir-Fe_2_O_3_ (62.0 mV dec^−1^), 2Ir-Fe_2_O_3_ (118.5 mV dec^−1^), and commercial IrO_2_ (70.3 mV dec^−1^) (Fig. 3B). Electrochemical impedance spectroscopy (EIS) at 1.5 V revealed that Ir incorporation significantly enhanced the conductivity of Fe_2_O_3_. Among the tested catalysts, 12Ir-Fe_2_O_3_ exhibited the lowest charge transfer resistance (fig. S15), indicating superior OER kinetics compared to 6Ir-Fe_2_O_3_, 2Ir-Fe_2_O_3_, pristine Fe_2_O_3_, and commercial IrO_2_. Moreover, compared with the 2Ir-Fe_2_O_3_ catalyst, the 12Ir-Fe_2_O_3_ catalyst exhibited a pronounced pH-dependent OER activity, suggesting enhanced reaction kinetics and a potentially distinct reaction mechanism (fig. S16). Together, these findings suggest a significant enhancement in the acidic OER activity as the surface Ir transitions from single atoms to atomic patches.

To assess the intrinsic catalytic activities of the materials, we determined the mass-specific activity by considering the total content of Ir and the turnover frequency (TOF) at each Ir site (Fig. 3C). The mass-specific activity of 12Ir-Fe_2_O_3_ at an overpotential (η) of 240 mV reaches 1599 A gIr^−1^, surpassing that of 6Ir-Fe_2_O_3_ (222.1 A gIr^−1^) and 2Ir-Fe_2_O_3_ (86.1 A gIr^−1^) by nearly 7 and 18 times, respectively. Notably, this activity exceeds that of commercial IrO_2_ by two orders of magnitude, measuring at 4.3 A gIr^−1^. Furthermore, the 12Ir-Fe_2_O_3_ catalyst displays the highest TOF of 0.795 s^−1^ at η = 240 mV, significantly surpassing that of 6Ir-Fe_2_O_3_ (0.11 s^−1^), 2Ir-Fe_2_O_3_ (0.043 s^−1^), and commercial IrO_2_ (0.002 s^−1^). To further elucidate the underlying reasons for the exceptional OER activity of 12Ir-Fe_2_O_3_, we first conducted electrochemical double-layer capacitance (*C*_dl_) tests to determine the electrochemically active surface area (ECSA) and roughness factor (*R*_f_) for activity normalization (fig. S17). In comparison to 6Ir-Fe_2_O_3_ and 2Ir-Fe_2_O_3_, 12Ir-Fe_2_O_3_ manifests the highest *C*_dl_ and ECSA, suggesting a progressive increase in the density of active sites with a higher Ir content. Despite the increase in Ir content correlating with a higher active site density, the specific activity, when normalized to the ECSA, still follows the order of 12Ir-Fe_2_O_3_ > 6Ir-Fe_2_O_3_ > 2Ir-Fe_2_O_3_. This observation suggests an enhancement in intrinsic OER activity as Ir transitions from single atoms to atomic patches, potentially due to the emergence of a previously unidentified catalytic mechanism. Forming Ir atomic patches on the carrier surface not only enhances the intrinsic OER activity of the noble metal Ir but also reduces Ir usage by minimizing internal Ir. It represents an effective approach to enable the widespread application of Ir in OER processes.

### Evaluation of catalyst stability

Although attaining high OER activity is crucial, stability may play an even more pivotal role in practical water-splitting applications. Thus, we subsequently evaluated the OER stability of the 12Ir-Fe_2_O_3_ catalyst under constant current density (10 mA cm^−2^) in various acidic electrolytes, including phosphoric acid (H_3_PO_4_), oxalic acid (H_2_C_2_O_4_), nitric acid (HNO_3_), sulfuric acid (H_2_SO_4_), perchloric acid (HClO_4_), and a mixed acid electrolyte of H_2_SO_4_ and HClO_4_. To enable a comparative assessment of OER stability among different catalysts, the acidity level of the electrolyte used was set at pH = 1 or 0. As depicted in Fig. 3D, the 12Ir-Fe_2_O_3_ catalyst demonstrates remarkable stability in OER under diverse acidic electrolyte conditions. In a highly acidic environment with a pH of 1 using the HClO_4_ electrolyte known for its strong oxidization properties, the 12Ir-Fe_2_O_3_ catalyst manifests stable operation for over 1500 hours (more than 2 months). In contrast, the 2Ir-Fe_2_O_3_ catalyst exhibits considerably shorter stability, operating for no more than 20 hours under similar conditions. Notably, the 12Ir-Fe_2_O_3_ catalyst could even operate stably for more than 2 months in a highly acidic pH = 0 HClO_4_ electrolyte, with a corresponding overpotential increase of only 30 mV observed at a current density of 10 mA cm^−2^ over a 1600-hour test period. The average overpotential increase for the 12Ir-Fe_2_O_3_ catalyst is 0.019 mV hour^−1^, indicating a rate more than 4000 times slower than that observed for the 2Ir-Fe_2_O_3_ catalyst (78.95 mV hour^−1^) (Fig. 3D). In fig. S18, a narrower *y*-axis range is used to improve the visibility of variations in the stability curves. The observed fluctuations are likely due to bubble formation during the OER. Nevertheless, from an overall perspective, the 12Ir-Fe_2_O_3_ catalyst exhibits excellent long-term stability. In addition to evaluating the long-term stability of the 12Ir-Fe_2_O_3_ catalyst in the HClO_4_ electrolyte, we also performed durability tests in other acidic media, such as H_2_SO_4_. As shown in fig. S19, the 12Ir-Fe_2_O_3_ catalyst remained stable for over 1 month (800 hours) in H_2_SO_4_, further demonstrating its long-term stability across a broad range of acidic electrolytes. The 12Ir-Fe_2_O_3_ catalyst exhibits negligible overpotential growth during long-term operation in strongly acidic electrolytes, indicating that lattice-confined Ir atomic patches maintain stable Ir─O bonding motifs under prolonged potential cycling, thereby preserving a robust and durable active site configuration. These findings suggest that the transition of Ir on the support, from Ir single atoms to Ir atomic patches, not only enhances its intrinsic activity but also markedly boosts its stability in acidic electrolytes. When considering both activity and stability as comparative metrics (*41*–*54*), the 12Ir-Fe_2_O_3_ catalyst demonstrates superior performance in acidic OER compared to analogous precious metal catalysts, even at low precious metal loadings (Fig. 3E and table S2).

To monitor the stability of 12Ir-Fe_2_O_3_ and 2Ir-Fe_2_O_3_ catalysts in HClO_4_ at pH = 0, we measured the remaining metal content in the catalyst using ICP-OES during the acidic OER (fig. S20). For the 12Ir-Fe_2_O_3_ catalyst, there was a notable increase in the dissolution of Ir and Fe elements in the acidic electrolyte within the initial 100 hours, followed by relatively stable levels thereafter. Even after 1600 hours of stability testing, over 99% of the Ir remained in the catalyst, highlighting that, although unprotected Fe_2_O_3_ initially dissolves, the Ir-covered regions remain stable and functional under acidic electrolysis conditions. In stark contrast, in the case of the 2Ir-Fe_2_O_3_ catalyst, the dissolution of Ir and Fe in the acidic electrolyte increases during the OER. This observed trend in dissolved contents correlates with variations in the catalyst’s electrochemical stability, suggesting that the formation of the atomic patch structure contributes to inhibition of dissolution. On the basis of the ICP-OES results, the stability number (*S*-number) of the catalysts was estimated (see Materials and Methods). The *S*-number of 12Ir-Fe_2_O_3_ at 1600 hours is 1.43 × 10^7^, outperforming rutile IrO_2_ (~10^6^; Alfa Aesar) (*55*) and other reported IrO_2_ (~10^5^; Sigma-Aldrich) (*42*), further demonstrating that 12Ir-Fe_2_O_3_ has excellent stability. Furthermore, the catalytic 12Ir-Fe_2_O_3_ was systematically characterized after long-term OER operation. The results show that its overall structure and properties remained largely unchanged (figs. S21 to S23). Notably, the Ir atom patches were essentially preserved in terms of size distribution, surface coverage, and crystallinity compared with the as-prepared catalyst. The remarkable durability of 12Ir-Fe_2_O_3_ under acidic conditions over 2Ir-Fe_2_O_3_ underscores the pivotal role of the Ir atomic patches. In addition, we assessed the catalyst’s performance in a proton exchange membrane water electrolyzer, where it also demonstrated impressive efficiency. As shown in Fig. 3 (F and G), the system achieves a current density of 1000 mA cm^−2^ at a cell voltage of only 1.8 V (without *iR* correction). Notably, after *iR* correction, the device exhibits activity that ranks among the highest reported for PEM electrolyzers to date (fig. S24). Further improvements in the activity of the above electrolyzers may be achieved through optimization of other components, including the substrate, membrane, cell geometry, and operating temperature; however, these factors are beyond the scope of this study. Furthermore, the device demonstrates stable performance for over 300 hours under continuous flow conditions (Fig. 3H). The long-term stability test in the PEM water electrolyzer further demonstrates that the 12Ir-Fe_2_O_3_ catalyst does not degrade the proton exchange membrane through metal ion dissolution, even under high current density (1000 mA cm^−2^) and acidic conditions. Meanwhile, the PEM device using 12Ir-Fe_2_O_3_ as the anode catalyst exhibits superior activity compared to the device using commercial IrO_2_ (fig. S25). These results strongly highlight the potential of this catalyst for large-scale industrial water electrolysis applications.

### Mechanistic investigation into activity

**Fig. 4.**
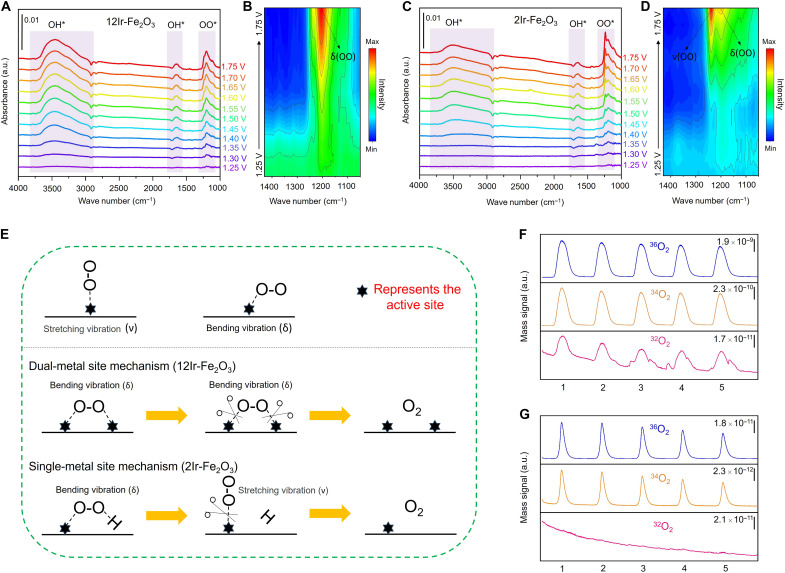
OER mechanism elucidation via in situ characterization. (**A**) In situ ATR-SEIRAS spectra (4000 to 1000 cm^−1^) of 12Ir-Fe_2_O_3_ under various potentials during OER; black vertical lines indicate the absorbance scale. (**B**) 2D color map of O─O bond vibration intensity derived from (A); simplified OER potential profile (black arrows) shown on the left axis. (**C**) In situ ATR-SEIRAS spectra of 2Ir-Fe_2_O_3_ under various potentials during OER; black vertical lines indicate the absorbance scale. (**D**) 2D color map of O─O bond vibration intensity derived from (C); simplified OER potential profile (black arrows) shown on the left axis. (**E**) Schematic diagrams of the vibration forms of the O─O bond during oxygen evolution according to the dual-metal site mechanism and the single-metal site mechanism, respectively. (**F** and **G**) DEMS signals of O_2_ products for (F) 12Ir-Fe_2_O_3_ and (G) 2Ir-Fe_2_O_3_ in the electrolyte using H_2_^18^O as the solvent during five cycles of CV in the potential range of 1.25 to 1.65 V versus RHE at a scan rate of 5 mV s^−1^. The black vertical lines on the top of the spectra in (F) and (G) show the scale of mass signals.

To elucidate the mechanism underlying the enhanced OER activity observed when transitioning from Ir single atoms to Ir atomic patches, we used in situ attenuated total reflectance surface-enhanced infrared absorption spectroscopy (ATR-SEIRAS) and operando differential electrochemical mass spectrometry (DEMS) to dynamically monitor the OER process on the surfaces of 12Ir-Fe_2_O_3_ and 2Ir-Fe_2_O_3_ catalysts (figs. S26 and S27). It is worth noting that IR spectroscopy and mass spectrometry are highly sensitive to the target substances, allowing these techniques to effectively overcome the challenge of low product detection resulting from sluggish catalyst kinetics. The reaction intermediates on the surface of 12Ir-Fe_2_O_3_ and 2Ir-Fe_2_O_3_ catalysts were detected via in situ ATR-SEIRAS over a potential range of 1.25 to 1.75 V under OER conditions. At 1.25 V, no discernible IR peak was evident in both 12Ir-Fe_2_O_3_ and 2Ir-Fe_2_O_3_ catalysts. Above 1.30 V, the peaks ranging from 2900 to 3800 cm^−1^ and from 1600 to 1800 cm^−1^ were detected (Fig. 4, A and C), indicating the presence of stretching (ν) and bending (δ) vibrations characteristic of surface OH* species (*56*, *57*). In addition, a pair of peaks ranging from 1100 to 1300 cm^−1^ were observed above 1.30 V for both the 12Ir-Fe_2_O_3_ and 2Ir-Fe_2_O_3_ catalysts, attributed to the O─O bond vibration of OO* species (*58*–*60*). To delve deeper into the interaction between the OO* species and active Ir sites, we compared the disparity in the IR spectra of 12Ir-Fe_2_O_3_ and 2Ir-Fe_2_O_3_ within the range of 1000 to 1450 cm^−1^ (Fig. 4, B and D, and fig. S28). At 1.30 V, the weak peaks corresponding to the δ and ν vibrations of the OO* species emerged at 1204 and 1239 cm^−1^ in 12Ir-Fe_2_O_3_, respectively (Fig. 4A). With increasing applied potential, the intensity of the peak corresponding to the δ vibration gradually rises, whereas that of the peak associated with the ν vibration remains relatively stable (fig. S28). In contrast, for 2Ir-Fe_2_O_3_, the intensity of the peak associated with the δ vibration remains relatively stable with increasing applied potential, whereas the intensity of the peak linked to the ν vibration gradually increases. This starkly contrasting trend directly suggests distinct configurations of active Ir sites between the 12Ir-Fe_2_O_3_ and 2Ir-Fe_2_O_3_ catalysts. Figure 4E illustrates the vibrational dynamics of OO* species at the active site during the oxygen evolution process, delineating between the dual-metal site (Fig. 4E, middle) and single-metal site (Fig. 4E, bottom) mechanisms via schematic representations of their respective configurations. Given that the OO* species predominantly undergoes δ vibrations on the surface of 12Ir-Fe_2_O_3_ (fig. S28), it follows that the active site on 12Ir-Fe_2_O_3_ with its atomic patch structure during the OER process, is likely dual-Ir atoms. By contrast, because the vibration of OO* species on the 2Ir-Fe_2_O_3_ surface shifts from δ to ν (fig. S28), it can be inferred that the active site during the OER process is the single Ir atom. Given the complexity of the OER process and the overlapping vibrational signatures of surface intermediates, ATR-SEIRAS alone cannot fully exclude minor *OOH contributions. Nevertheless, the distinct O─O vibrational behavior observed for 12Ir-Fe_2_O_3_ versus 2Ir-Fe_2_O_3_, combined with their different atomic configurations, strongly supports a dual-metal site pathway for the former. This interpretation is further corroborated by operando DEMS and theoretical calculations discussed below.

To further verify that the 12Ir-Fe_2_O_3_ catalyst, characterized by an atomic patch structure, predominantly follows a dual-metal site mechanism during the OER process, we conducted operando DEMS coupled with isotope labeling measurements. We implemented a two-step DEMS experiment, using H_2_^18^O and H_2_^16^O as supporting solutions (pH = 1, HClO_4_), respectively. In the initial step, 12Ir-Fe_2_O_3_ and 2Ir-Fe_2_O_3_ were deposited onto Au working electrodes and underwent five cyclic voltammetry (CV) cycles [1.25 to 1.65 V versus reversible hydrogen electrode (RHE)] in the H_2_^18^O electrolyte. Given that both the 12Ir-Fe_2_O_3_ and 2Ir-Fe_2_O_3_ catalysts host surface-adsorbed oxygen species (O_ads_) (*33*, *61*–*63*), if the catalyst evolves oxygen according to the dual-metal site mechanism, then ^16^O_ads_ on two neighboring Ir sites may combine to produce ^32^O_2_. Figure 4 (F and G) displays the recorded mass signals of the OER gaseous products. The 12Ir-Fe_2_O_3_ consistently generated ^32^O_2_, ^34^O_2_, and ^36^O_2_ in each CV cycle (fig. S29). In contrast, the 2Ir-Fe_2_O_3_ catalyst with isolated Ir atoms produced ^34^O_2_ and ^36^O_2_ (fig. S30). It is worth noting that the ^32^O_2_ product signal was indistinguishable from the background noise as the OER proceeded via the single-metal site pathway. In the second step, the catalyst labeled with ^18^O in the first step was thoroughly washed with a substantial quantity of water (H_2_^16^O), followed by operation in the H_2_^16^O electrolyte. If the dual-metal site mechanism were operational in the case of 12Ir-Fe_2_O_3_, it is likely that the residual surface adsorbates, containing ^18^O, would coalesce to produce the ^36^O_2_ product (fig. S31). This contrasts with the absence of detectable ^36^O_2_ product in the case of 2Ir-Fe_2_O_3_, as illustrated in fig. S32. The operando DEMS findings corroborated our predictions, as depicted in fig. S33. Together, both in situ ATR-IR and operando DEMS validated that 12Ir-Fe_2_O_3_, with its atomic patch structure, predominantly proceeds the dual-metal site mechanism in the OER process. Conversely, 2Ir-Fe_2_O_3_, characterized by isolated single atoms, tends to adhere to the single-metal site mechanism in the OER process. This discrepancy in mechanism is the primary factor dictating the observed difference in OER activity.

**Fig. 5.**
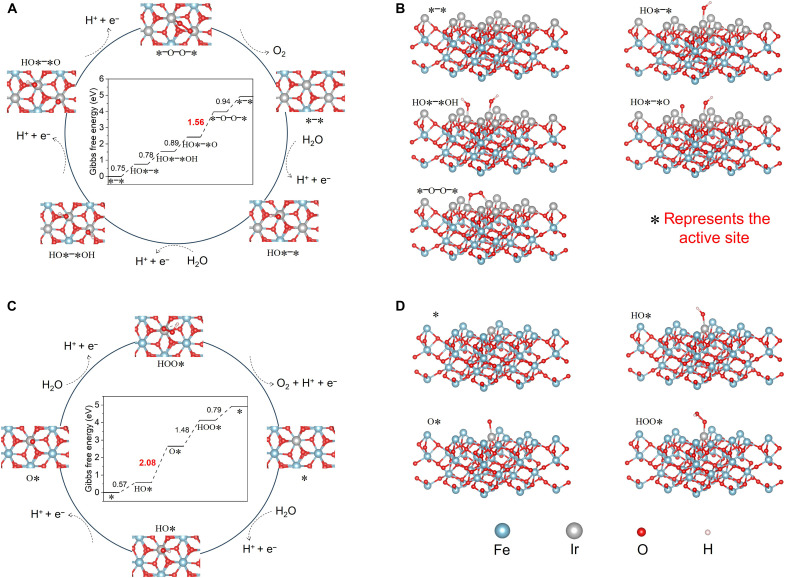
OER mechanism elucidation through theoretical calculations. (**A** and **B**) Top view (A) and side view (B) of the OER pathway based on the dual-metal site mechanism on the Ir atomic patch catalyst. The inset in (A) depicts the calculated energy profile, where the initial Ir atomic patch is used as the reference for the energy (in eV). (**C** and **D**) Top view (C) and side view (D) of the OER pathway based on the single-metal site mechanism on the Ir single-atom catalyst. The inset in (C) shows the calculated energy landscape, with the initial Ir single atom serving as the reference for the energy (in eV).

To complement and reinforce the findings from the aforementioned in situ experiments, first-principles calculations were conducted to provide additional insights into the OER mechanism of 12Ir-Fe_2_O_3_. In our experiments, XRD analysis confirmed that the elevated content of Ir in the 12Ir-Fe_2_O_3_ sample predominantly localized on the external surface of α-Fe_2_O_3_. In addition, aberration -corrected HAADF-STEM revealed abundant island-like patches of Ir atoms distributed across the α-Fe_2_O_3_(001) surface. Furthermore, before theoretical calculations, in situ Raman spectroscopy (fig. S34) was used to probe the evolution of surface species during OER. The potential-dependent shift of Ir─O vibrational modes, in contrast to the invariant Fe─O features, indicates that Ir-centered sites dominate the catalytic process under acidic conditions. According to these results, we used α-Fe_2_O_3_ with an exposed (001) surface as the fundamental computational model. Subsequently, an atomic patch was generated by substituting all Fe atoms with three-coordinated oxygen atoms on its outer surface with Ir atoms (fig. S35). Figure 5 (A and B) depicts the OER mechanism on the Ir site of the Ir atomic patch catalyst, featuring two active sites and encompassing five elementary steps. The free energy change for each elementary step is included as an inset in Fig. 5A. First, a water molecule undergoes dissociation from an Ir atom, releasing H^+^ ions and leading to the formation of *OH groups. This step has an endothermic nature, characterized by a Gibbs free energy change of 0.75 eV. Next, another water molecule experiences dissociation, releasing H^+^ ions on the adjacent Ir atom, thereby resulting in the formation of two neighboring *OH groups, accompanied by a free energy change of 0.78 eV. Following this, two consecutive proton-coupled electron transfer steps take place, during which the protons from two adjacent *OH groups are released into the solution, accompanied by the O─O coupling on two adjacent Ir active sites and the formation of *O─O species. Last, *O─O desorbs from the catalyst surface and reverts to the initial state, accompanied by a free energy change of 0.94 eV. Overall, the O─O coupling in the dual-metal site mechanism emerges as the rate-determining step, characterized by the largest free energy change of 1.56 eV. To further rule out the possibility that OER on the Ir atomic patch catalyst occurs via a single-metal site mechanism, we calculated the energy profiles associated with oxygen evolution using this mechanism, as shown in figs. S36 and S37. Our results indicate that the potential barrier of the single-metal site mechanism is 1.80 eV, representing a 0.24-eV increase compared to the dual-metal site mechanism. Thus, for atomic patch catalysts (12Ir-Fe_2_O_3_), the occurrence of the dual-metal site mechanism during the OER process is more probable than the single-metal site mechanism, corroborating the results of the in situ/operando spectroscopy characterizations.

To unfold why the atomic array catalyst (12Ir-Fe_2_O_3_) manifests greater activity than the single-atom catalyst (2Ir-Fe_2_O_3_), we also calculated the energy profiles for the single-atom catalyst during the OER process (Fig. 5, C and D, and fig. S38). The calculations signify that the rate-limiting step in the oxygen evolution process is the removal of the proton from *OH group, with a corresponding Gibbs free energy change of 2.08 eV. Compared to the energy barrier of the atomic patch catalyst, the single-atom catalyst exhibits a higher energy barrier, demonstrating that the atomic patch catalyst has higher oxygen evolution activity. The difference in activity between atomic patch catalysts and single-atom catalysts can be better understood through their electronic band structures. As shown in fig. S39, the Ir atoms in the atomic patch catalyst (−1.49 eV) and in the single-atom catalyst (−1.73 eV) display different d-band centers. According to the d-band theory, transition metals with higher d-band centers have fewer occupied antibonding states with adsorbates, resulting in stronger bonds between the metal atoms and adsorbates. Compared to Ir single-atom catalysts, the higher OER activity of Ir atomic patch catalysts may stem from their stronger binding affinity with oxygen-containing intermediates (*64*). Thus, the formation of the atomic patch structure alters the electronic band structure, influencing the surface binding characteristics and catalytic activity of the catalyst (*65*–*67*).

### Mechanistic scrutiny of stability

**Fig. 6.**
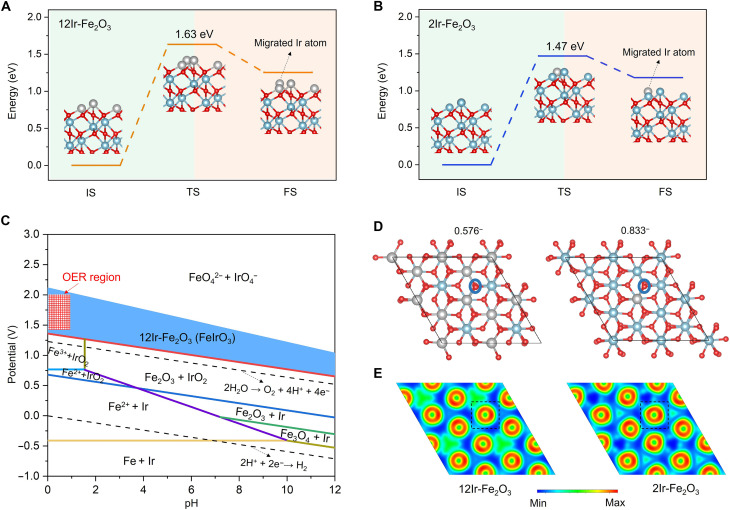
Theoretical investigation into stability. (**A** and **B**) Calculated migration energies of Ir atoms on (A) 12Ir-Fe_2_O_3_ and (B) 2Ir-Fe_2_O_3_. The inset structures represent the initial state (IS), transition state (TS), and final state (FS), respectively. Light blue, gray, and red spheres represent Fe, Ir, and O atoms, respectively. (**C**) Surface Pourbaix diagram for the 12Ir-Fe_2_O_3_ obtained from the calculations. As the molar ratio of the outermost Ir atoms and the second outermost Fe atoms is 1:1, FeIrO_3_ is taken as the surface structure of 12Ir-Fe_2_O_3_. (**D**) Charge numbers of surface oxygen atoms (marked with blue circles) based on the Bader charge calculation results. The structural models on the left and right depict the surface structure of 12Ir-Fe_2_O_3_ and 2Ir-Fe_2_O_3_, respectively. (**E**) ELF maps of surface oxygen atoms, corresponding to surface structure model in (D).

We now turn our attention to understand the experimental stability results by using first-principles calculations to scrutinize how the formation of Ir atomic patches enhances catalyst stability in acidic electrolytes. α-Fe_2_O_3_ with an exposed (001) surface remains the basis for computational models of stability. We used two specific structure models in the calculation: the atomic patch catalyst, where all surface Fe atoms are replaced by Ir atoms (12Ir-Fe_2_O_3_), and the single-atom catalyst, where one surface Fe atom is replaced by an Ir atom (2Ir-Fe_2_O_3_). Notably, these structures are consistent with those used in activity calculations above. Given that catalyst dissolution in acidic electrolytes involves the migration of surface-active Ir atoms, we assessed the stability of corresponding catalysts by calculating the migration energies of these lattice Ir atoms. For the Ir atom patch catalyst, we proposed the migration process of Ir atoms on the model surface, as depicted in Fig. 6A and fig. S40. This process involves Ir atoms moving away from the surface via a transition state with an energy barrier of 1.63 eV. Subsequently, we determined the migration energy of Ir atoms on the surface in the Ir single-atom model (2Ir-Fe_2_O_3_). The migration energy of the Ir single atoms is established at 1.47 eV (Fig. 6B and fig. S41), indicating a higher likelihood of surface migration compared to Ir sites in atomic patch catalysts. It suggests that Ir sites within the atomic patches display greater stability than Ir single atoms under acidic OER conditions. Moreover, this result underpins the experimental observations (Fig. 3D) that the formation of the Ir atomic patch structure effectively hinders catalyst dissolution, thereby enhancing the stability of the Ir-based catalyst.

To further understand the enhanced acid resistance of Ir atomic patch catalysts (12Ir-Fe_2_O_3_) under electrolysis conditions, we calculated their corresponding surface Pourbaix diagrams. Because the experimental results confirmed that the Ir atomic patch is fully exposed and dispersed on the (001) surface of Fe_2_O_3_ without penetrating into the bulk, we considered only Ir atoms in the outermost layer while treating Fe_2_O_3_ as an inner layer during the calculation of the Pourbaix diagram. Thus, on the basis of the basic model of Fe_2_O_3_, we used the structural model of 12Ir-Fe_2_O_3_ as the catalyst for the Ir atomic patches. In addition, as the Ir atomic patch is in the outermost layer of the catalyst, the dissolution of 12Ir-Fe_2_O_3_ will initially involve the outer Ir atoms rather than the Fe atoms. Figure 6C illustrates the phase stability of 12Ir-Fe_2_O_3_ in Pourbaix diagrams, showing its behavior in an aqueous environment concerning dissolved and solid phases. In our acidic OER tests, the operating voltage ranges from 1.4 to 2.0 V, and the electrolyte pH is between 0 and 1. Therefore, electrolysis occurs within these boundaries, as indicated by the red rectangle in Fig. 6C. This operating window falls within the stable 12Ir-Fe_2_O_3_ phase, theoretically rationalizing the ultrahigh stability of the Ir atomic patch catalyst under strongly acidic conditions. Furthermore, we scrutinized the electron distribution on the surface of both Ir atomic patch catalysts and Ir single-atom catalysts to deepen our understanding of surface structure stability. The Bader charge calculations reveal that the oxygen atoms adjacent to Ir atoms in the Ir atomic patch catalyst have fewer electrons (0.576^−^) compared to those in the Ir single-atom catalyst (0.833^−^) (Fig. 6D). This suggests that more electrons are transferred from Ir in the Ir single-atom catalyst to the neighboring oxygen. This transfer results in a high local electron concentration, leading to increased local stress and decreased stability of the surface structure. The electron localization function (ELF) calculations further unravel that the electron distribution around oxygen atoms on the surface of the Ir atomic patch catalyst is more uniform (inside a square-shaped box on the left) than that on the Ir single-atom catalyst (inside a square-shaped box on the right; Fig. 6E). This insinuates a lower degree of electron localization on the Ir atomic patch catalyst, resulting in higher surface structure stability. Together, the results of Pourbaix diagram, Bader charge analysis, and ELF calculations collectively substantiate that the formation of Ir atomic patch effectively inhibits catalyst dissolution in acidic electrolytes, thereby demonstrating ultrahigh stability.

## DISCUSSION

In summary, we report overcoming the activity-stability trade-off in acidic water oxidation by invoking metal atom patches with ample dual-metal sites, achieved through a facile lattice confinement strategy. Notably, the judiciously designed Ir atom patch favors the OPM mechanism and displays both superior activity and stability under acidic conditions. It attains a current density of 10 mA cm^−2^ with a markedly low overpotential of 198 mV and demonstrates a mass activity two orders of magnitude higher than commercial IrO_2_. This enhanced mass activity could greatly reduce the amount of iridium needed for large-scale applications. As revealed by in situ characterization and DFT calculations, the OER on the Ir atom patch proceeds via a dual-active site mechanism, with direct O─O radical coupling as the key step. The lattice confinement-induced unique pathway enables Ir atom patches to exceed the activity limitations of traditional single-site mechanisms (i.e., AEM and LOM). In addition, the catalyst demonstrates stable operation in strong acidic electrolytes for over 2 months (1500 hours at pH = 1 and 1600 hours at pH = 0). Theoretical stability calculations unveil that the atomic patch structure effectively inhibits catalyst dissolution in acidic electrolytes. This work highlights an effective strategy for concurrently enhancing activity and stability by building the active site landscape via lattice confinement, representing a notable advancement in reducing precious metal consumption and promoting sustainable energy development.

## MATERIALS AND METHODS

### Synthesis of α-Fe_2_O_3_ nanosheets

Preparation of α-Fe_2_O_3_ nanosheets using a modified solvothermal method as previously reported (*34*). In a typical procedure, 1.01 mmol of FeCl_3_·6H_2_O was dissolved in 10.0 ml of ethanol under vigorous stirring, and 0.7 ml of water was added. Once completely dissolved, 9.76 mmol of sodium acetate was added while stirring. The mixture was then sealed in a 25-ml Teflon-lined stainless steel autoclave and maintained at 180°C for 12 hours to allow for solvothermal crystallization. After cooling to ambient temperature, the resulting solid products were washed several times with deionized water, separated by vacuum filtration, and dried overnight in an oven at 60°C.

### Synthesis of Ir-Fe_2_O_3_ nanosheets

In a typical procedure, 0.62 mmol of Fe_2_O_3_ nanosheets were dispersed in 20 ml of water and sonicated for 25 min. A specific amount of H_2_IrCl_6_·*x*H_2_O was dissolved in another 20 ml of water and then injected into the Fe_2_O_3_ suspension at room temperature under vigorous stirring. The reaction was allowed to proceed for 12 hours. The resulting mixture was first vacuum filtered to remove some of water. The remaining sample was then dried at 80°C for 12 hours and calcined in air at 350°C for 1 hour. The concentrations of H_2_IrCl_6_ of 1.06, 5.84, and 19.42 mM were used for 2Ir-Fe_2_O_3_, 6Ir-Fe_2_O_3_, and 12Ir-Fe_2_O_3_, respectively, resulting in Ir contents of 1.8, 6.3, and 12.1%, as determined by ICP-OES analysis. When the concentration of the H_2_IrCl_6_ precursor exceeds 19.42 mM, the filtrate after vacuum filtration exhibits a distinct Ir ion color, indicating that the Fe_2_O_3_ support has reached its maximum Ir uptake capacity. Accordingly, the composition corresponding to 12Ir-Fe_2_O_3_ represents the upper limit of effective lattice incorporation under our synthesis conditions.

### Structural and morphological characterization

SEM images were obtained using field-emission scanning electron microscopy (FESEM; JSM-6700F). TEM, HAADF-STEM, and EDS elemental mapping were performed on a transmission electron microscope equipped with energy-dispersive x-ray spectroscopy (HRTEM/EDX; JEM-2100F, 200 kV). Atomic-resolution TEM and HAADF-STEM images were acquired using a JEOL ARM300F microscope with a probe-forming aberration corrector, operated at 300 kV. XPS data were collected using XXPS with an Mg-Kα source (XSAM800 XPS). XRD data were gathered using a powder diffractometer (Bruker D8 Advance) with Bragg-Brentano geometry for 2θ values ranging from 5° to 80°. XAFS measurements were performed at the XAFCA beamline of the Singapore Synchrotron Light Source (SSLS) at room temperature and ambient pressure. Samples with high metal content were measured in transmission mode, whereas those with low metal content were measured in fluorescence mode, using a metal foil as a reference. Data processing and EXAFS fitting were conducted using the Demeter program.

### Electrochemical measurements

Electrochemical tests were conducted in a three-electrode electrochemical cell using either 0.1 or 1 M HClO_4_ as the electrolyte, with an Ag/AgCl reference electrode and a Pt mesh counter electrode. Catalyst dispersions were prepared by mixing 1 ml of ethanol, 25 μl of Nafion solution (5 wt %, Du Pont), 2 mg of XC-72, and 4 mg of the catalyst, followed by sonication. The addition of XC-72 aims to enhance the catalyst’s conductivity, thereby maximizing its observed OER activity, without altering its intrinsic catalytic properties. To ensure comparability, the same total catalyst mass (4 mg) was used to prepare all inks, including those of 12Ir-Fe_2_O_3_ and commercial IrO_2_. Because Ir in 12Ir-Fe_2_O_3_ is predominantly located at the outer surface of the support, most Ir atoms are electrochemically accessible, whereas in nanoparticulate IrO_2_ only surface Ir atoms contribute directly to catalysis. Subsequently, 25 μl of the ink was evenly loaded onto a glassy carbon electrode (5 mm in diameter, 0.196 cm^2^), serving as the working electrode. The catalyst loading was selected on the basis of values reported in the previous literature (*14*, *26*, *51*). This loading also allowed for the formation of a uniform and flat catalyst layer on the glassy carbon electrode, without ink overflow during the preparation process. Electrochemical measurements were performed using a rotating disk electrode (RDE) in an O_2_-saturated 0.1 or 1 M HClO_4_ electrolyte at 25°C, with a scan rate of 5 mV s^−1^ at 1600 rpm. Before LSV testing, the catalyst was scanned repeatedly from 0.45 to 0.95 V in 0.1 or 1 M HClO_4_ until a stable voltammetry curve was achieved. To convert potentials to the RHE scale, the Ag/AgCl reference electrode was calibrated in H_2_-saturated 0.1 or 1 M HClO_4_ by measuring hydrogen oxidation/evolution on a platinum mesh electrode (figs. S42 and S43). EIS tests were conducted at various applied potentials versus RHE in the frequency range of 0.01 to 50,000 Hz. Postmeasurement *iR* compensation was performed using potential-EIS to measure uncompensated resistance. The potentials were corrected for solution resistance using the equation: *E*_*iR*-corrected_ = *E* − *iR*_s_, where *R*_s_ (7.2 Ω) is the uncompensated solution resistance measured by high-frequency ac impedance in 1 M HClO_4_. To determine the double-layer capacitance (*C*_dl_), CV was performed at various scan rates (2, 4, 8 mV s^−1^, etc.) between 1.1 and 1.2 V (versus RHE).

The long-term stability of the catalysts in various acidic electrolytes was tested on carbon fiber paper (CFP) using chronopotentiometry. The inherent porous structure of carbon paper serves a similar function to the high-speed rotation of an RDE, facilitating rapid bubble release and thereby reducing mass transfer resistance. As shown in fig. S44, the Tafel slopes of the polarization curves for 12Ir-Fe_2_O_3_ supported on both glassy carbon and carbon paper are nearly identical, indicating comparable OER kinetics on the two substrates. To load the catalysts onto CFP, 3 mg of the catalyst was dispersed in a mixture of 1 ml of ethanol and 25 μl of Nafion solution (5 wt %, DuPont), forming a uniform ink with ultrasonic assistance. No additional carbon (XC-72) was introduced during the electrochemical test using carbon paper because its porous structure allows the deposited catalyst to fully contact the conductive substrate, eliminating the need for supplemental carbon. The ink was then applied to a 1-cm^2^ carbon paper surface using a micropipette and dried at room temperature, resulting in a final catalyst loading of 3 mg cm^−2^. All potentials are referenced to the RHE with *iR* compensation, where *i* is the measured current and *R* is the uncompensated resistance determined by EIS. The Faradaic efficiencies of O_2_ generated were measured as ~100% for 12Ir-Fe_2_O_3_ (fig. S45).

### PEMWE tests

The membrane electrode assembly (MEA) was prepared using the catalyst-coated membrane method. Catalyst dispersions were prepared as described above. Pt/C (20 wt %, MERCK) and synthesized 12Ir-Fe_2_O_3_ were spray-coated onto opposite sides of a Nafion-115 membrane (CHEMOURS) over a masked area of 2.25 cm by 2.25 cm. The mass loadings of the cathode and anode catalyst layers were ≤0.5 and ≤1.0 mg cm^−2^, respectively, determined by the mass difference before and after spray-coating. CFP (CFP-28 BC, SIGRACET) and titanium felt (MAINZ) were used as the gas diffusion layers for the cathode and anode, respectively. The MEA was then assembled into a proton exchange membrane water electrolyzer (MAINZ-Pt-PEM04). A 0.1 M HClO_4_ electrolyte was fed into the anode chamber, and electrolysis was conducted at 70° to 75°C. Electrochemical performance was evaluated using a potentiostat (CS315M, CORRTEST) at a scan rate of 5 mV s^−1^. Unless otherwise noted, all PEMWE results are reported without *iR* compensation. It is worth noting that, although current industrial PEM electrolysis typically uses pure water to minimize material costs, this leads to limited mass transport and high solution resistance, thereby increasing energy consumption. For more sustainable and cost-effective large-scale deployment, a comprehensive assessment of both pure water and acidic electrolytes is necessary. Our use of 0.1 M HClO_4_ is also consistent with prior fundamental studies, allowing for reliable comparison.

### Experimental calculation methods

The mass activity (*j*_mass activity_) of the catalysts is calculated by the following equation

$$j_{\text{mass activity}}=\frac{j_{\text{geo}\times S_{\text{geo}}}}{m_{\text{Ir}}}$$

where *j*_geo_ represents the geometric current density, *s*_geo_ denotes the geometric area, and *m*_Ir_ is the mass of Ir loaded onto the glassy carbon determined from ICP-OES results.

The TOF is calculated by the following equation

$$\text{TOF}=\frac{j_{\text{geo}\times S_{\text{geo}}}}{4F\times n}$$

where *j*_geo_ represents the geometric current density, *s*_geo_ is the geometric area of the glassy carbon electrode, 4 is the total number of electrons required for generating one O_2_ molecule, *F* is the Faraday’s constant (96,485.3 C mol^−1^), and *n* is the moles of Ir in the sample loaded on the electrode. It is worth noting that the TOF is calculated on the basis of the total molar amount of Ir loaded on the electrode. However, because the actual number of Ir atoms functioning as active sites is likely lower than the total Ir content, the true TOF may be higher than the calculated value.

The ECSA is calculated using the formula: ECSA = *C*_dl_/*C*_s_, where *C*_dl_ is the double-layer capacitance and *C*_s_ is the specific capacitance of the sample. In this study, we used a general specific capacitance value of *C*_s_ = 0.035 mF cm^−2^, based on the typical reported value (*14*). The double-layer capacitance, *C*_dl_, is determined by the equation *C*_dl_ = *i*_c_/ν, where *i*_c_ is the charging current and ν is the scan rate. A series of CV tests was performed in the non-Faradaic potential region (1.1 to 1.2 V relative to the RHE) at various scan rates (2, 4, 8, 12, 16, and 20 mV s^−1^). *C*_dl_ is obtained from the slope of the linear fit of the plot of *i*_c_ versus ν. The roughness factor was then calculated by dividing the ECSA by the geometric area of the electrode, which, in this study, was 0.196 cm^−2^. Considering that carbon was added to each catalyst in the electrochemical test, the ECSA calculated from double-layer capacitance may not accurately reflect the absolute electrochemically active area. However, because the same amount of carbon was used for all samples, the ECSA values reliably indicate the relative trend in active site density.

The *S*-number was calculated using the following equation

$$S=\frac{n_{O_{2}}}{n_{\text{Ir}\left(\text{dissolved}\right)}}$$

where $n_{O_{2}}$ is the molar amount of total oxygen evolved over a given period (calculated from the total charge), and *n*_Ir(dissolved)_ is the total dissolved iridium as measured by ICP-OES.

### In situ ATR-SEIRAS spectroscopy

For in situ ATR-SEIRAS measurements, a custom spectroelectrochemical cell featuring a three-electrode configuration was used. The catalyst, applied to an Au film, served as the working electrode, whereas a calibrated Ag/AgCl electrode and a platinum sheet functioned as the reference and counter electrodes, respectively. The ATR-SEIR analysis was conducted using a Bruker Vertex 70v spectrometer equipped with a liquid nitrogen–cooled mercury cadmium telluride (MCT) detector and an IR optical accessory (SPEC-I, Shanghai Yuanfang Tech.) configured for an optimized beam incidence angle of ~60° on the reflection plane of the working electrode. To minimize atmospheric interference, the interferometer and specular reflection assembly were purged with N_2_, mitigating H_2_O and CO_2_ signals. Spectra were recorded with a resolution of 8 cm^−1^, and each single beam spectrum acquisition last ~20 s. All spectra were presented in absorbance, defined as −log(*R*/*R*_0_), where *R* and *R*_0_ represented the sample and reference single beam spectra, respectively.

### Operando DEMS with isotope labeling

DEMS using the QAS100 instrument from Shanghai Linglu, in conjunction with a PARSTAT electrochemical workstation (VersaSTAT 3F), was used in a standard three-electrode electrochemical cell setup. A calibrated Ag/AgCl electrode served as the reference, whereas a platinum wire acted as the counter electrode. The working electrode comprised a gold film sputtered onto a porous polytetrafluoroethylene (PTFE) membrane, onto which the catalyst was sprayed and subsequently dried. The hydrophobic nature of the PTFE membrane allowed gas flow while preventing liquid ingress. To prevent potential damage to the mass spectrometer, a cold trap cooled with dry ice was positioned between the electrochemical cell and the vacuum chamber, effectively trapping water vapor. Isotope labeling studies used ~1 ml of 0.1 M HClO_4_ solution with H_2_^18^O as the solvent. Five consecutive CV cycles within the range of 1.15 to 1.65 V versus RHE at a scan rate of 5 mV s^−1^ were conducted to label the catalyst surface with ^18^O while simultaneously recording the mass signals of the gaseous products ^32^O_2_, ^34^O_2_, and ^36^O_2_. Subsequently, the labeled catalyst was thoroughly washed with abundant H_2_^16^O to remove any residual H_2_^18^O molecules. Following the wash, the ^18^O isotope–labeled catalyst was subjected to operation in 0.1 M HClO_4_ solution with H_2_^16^O as the solvent, and once again, the mass signals of the gaseous products ^32^O_2_, ^34^O_2_, and ^36^O_2_ were monitored.

### Computational details

All computations were conducted using spin-polarized DFT as implemented by the Vienna Ab initio Simulation Package (VASP) (*68*). Projector augmented wave (PAW) pseudopotentials were used to describe ionic potentials (*69*). The exchange-correlation energy was modeled using the Perdew-Burke-Ernzerhof (PBE) functional within the generalized gradient approximation (GGA) (*70*, *71*). During geometry relaxation, the energy cutoff was set to 400 eV, and the Brillouin zone was sampled using a 5 × 5 × 1 *k*-point mesh based on the Monkhorst-Pack scheme. The convergence criterion for the self-consistent field (SCF) iterations was 10^−5^ eV, and the force convergence criterion for atomic relaxation was 0.01 eV Å^−1^. A vacuum gap of 10 Å was maintained to prevent interactions between adjacent active sites.

The Gibbs free energy changes (Δ*G*) for each electrochemical elementary reaction were estimated using the following formula, using the theoretical hydrogen electrode model to simulate these OERs

$$\Delta G=\Delta E_{\text{ZPE}}+\Delta E+\Delta G_{\text{pH}}+\Delta G_{U}-T\Delta S$$

where Δ*E*_ZPE_ represents the difference in zero-point energy at 298.15 K, calculated from the vibrational frequencies of the products and reactants. The value of Δ*E* is derived from the total energies. The effects of electrode pH and applied voltage are corrected using the Δ*G*_pH_ and Δ*G*_U_ terms, respectively. Δ*S*, the entropy change, is obtained similarly to Δ*E*_ZPE_.

Accordingly, the Gibbs free energy changes for the water oxidation steps via the single-metal site mechanism were calculated using the following equations (*33*)

$$H_{2}O+{}^{*}\to {\text{OH}}^{*}+H^{+}+e^{-}$$

$${\text{OH}}^{*}\to O^{*}+H^{+}+e^{-}$$

$$O^{*}+H_{2}O\to {\text{OOH}}^{*}+H^{+}+e^{-}$$

$${\text{OOH}}^{*}\to O_{2}+{}^{*}+H^{+}+e^{-}$$

Furthermore, the Gibbs free energy changes for the water oxidation steps via the dual-metal site mechanism were calculated using the following equations

$$H_{2}O+{M_{1}}^{*}-{M_{2}}^{*}\to \text{OH}-M_{1}-{M_{2}}^{*}+H^{+}+e^{-}$$

$$\text{OH}-M_{1}-{M_{2}}^{*}+H_{2}O\to \text{OH}-M_{1}-M_{2}-\text{OH}+H^{+}+e^{-}$$

$$\text{OH}-M_{1}-M_{2}-\text{OH}\to O-M_{1}-M_{2}-\text{OH}+H^{+}+e^{-}$$

$$O-M_{1}-M_{2}-\text{OH}\to M_{1}-O-O-M_{2}+H^{+}+e^{-}$$

$$M_{1}-O-O-M_{2}\to {M_{1}}^{*}-{M_{2}}^{*}+O_{2}$$

To assess the stability of the Ir atom array during OER processes in an aqueous environment, we calculated the Pourbaix diagrams. These diagrams map stable phases as a function of pH and electrochemical potential. The thermodynamic data for the involved ions were obtained from ref. (*72*). In an aqueous medium at a given pH (−log[H^+^]) and potential (*E*), the following redox reaction occurs

$$\left[\text{Reactants}\right]+H_{2}O\Leftrightarrow \left[\text{Products}\right]+mH^{+}+ne^{-}$$

At equilibrium, the Gibbs free energy change (Δ*G*_rxn_) of this reaction can be related to *E* using the Nernst equation

$$\begin{array}{c} -\mathrm{nFE}=\Delta G_{\text{rxn}}=\Delta G_{\text{rxn}}^{0}+2.303\times \mathrm{RT}\times \\ \text{log}\frac{a_{\text{Reactants}}}{a_{\text{Products}}}-2.303\times \mathrm{RT}\times m\times \text{pH} \end{array}$$

where Δ$G_{\text{rxn}}^{0}$ is the Gibbs free energy change of the reaction under standard conditions, *F* is the Faraday’s constant, *R* is the gas constant, *T* is the temperature, and *a* is the activity coefficient. The most stable species in aqueous solutions can be determined by minimizing (Δ*G*_rxn_ + *nFE*) across all possible reactions under specific pH and applied potential conditions.
