# Supplementary material for: Breaking the activity-stability limit in acidic oxygen evolution reaction with dual-iridium active sites
Source: Sci Adv. 2026 Apr 15;12(16):eaee7510. doi: 10.1126/sciadv.aee7510 (PMC13082336; doi:10.1126/sciadv.aee7510)
Supplement: Supplementary file 1 — Figs. S1 to S45 Tables S1 and S2 [file sciadv.aee7510_sm.pdf]

Science Advances
AAAS

Supplementary Materials for

Breaking the activity-stability limit in acidic oxygen evolution reaction with dual-iridium active sites

Lei Tang *et al.*

Corresponding author: Yan Yan, yanyan@ahut.edu.cn; Yan Liu, liu_yan@a-star.edu.sg; Yijiang Liu, liuyijiang84@xtu.edu.cn; Javier Pérez-Ramírez, jpr@chem.ethz.ch; Zhiqun Lin, z.lin@nus.edu.sg

Sci. Adv. **12**, eaee7510 (2026)
DOI: 10.1126/sciadv.aee7510

This PDF file includes:

Figs. S1 to S45
Tables S1 and S2

Supplementary Figures

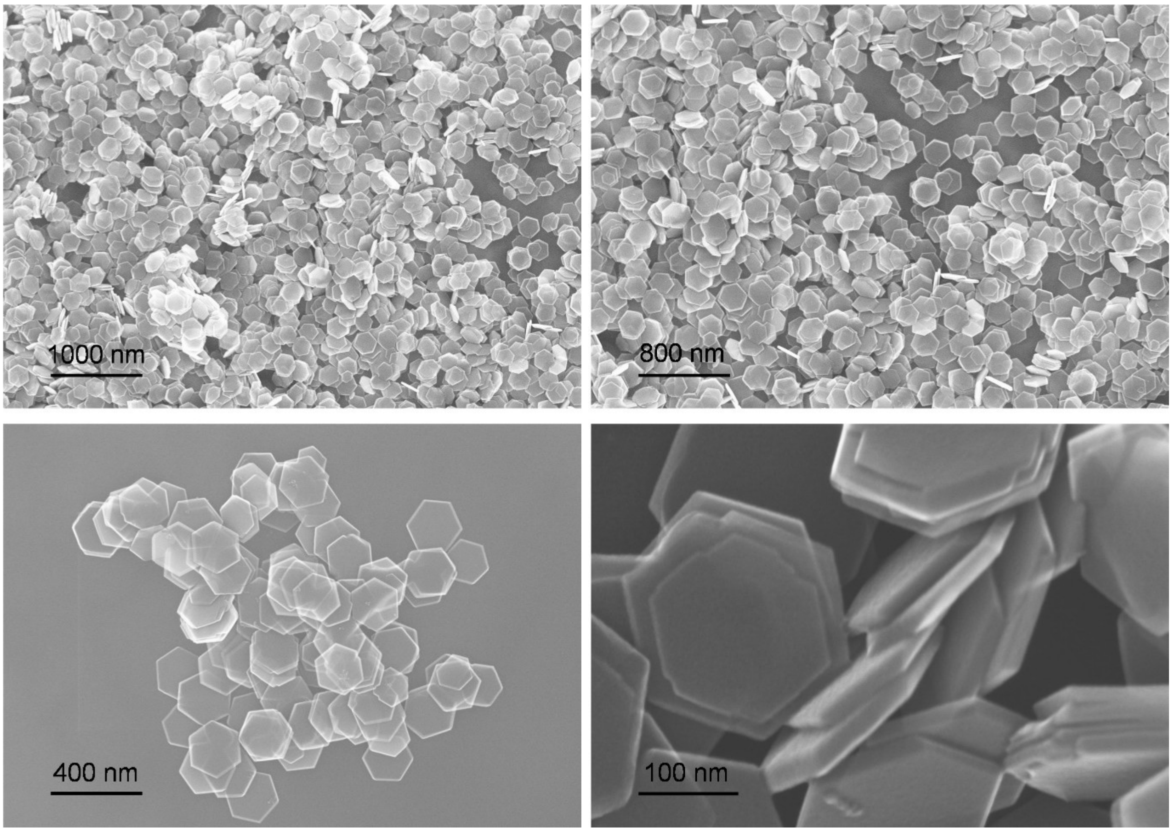

Fig. S1. Scanning electron microscope (SEM) images of α-Fe_2O_3 nanosheets at different magnifications.

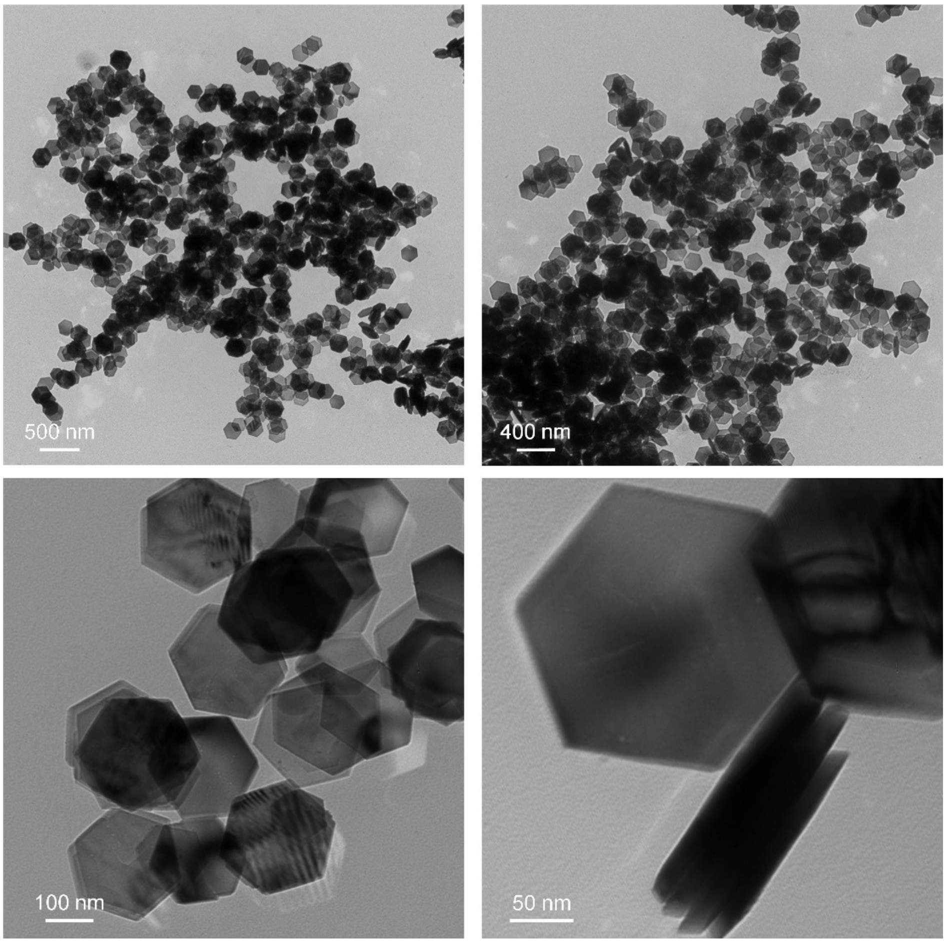

Fig. S2. Transmission electron microscopy (TEM) images of α-Fe_2O_3 nanosheets at different magnifications.

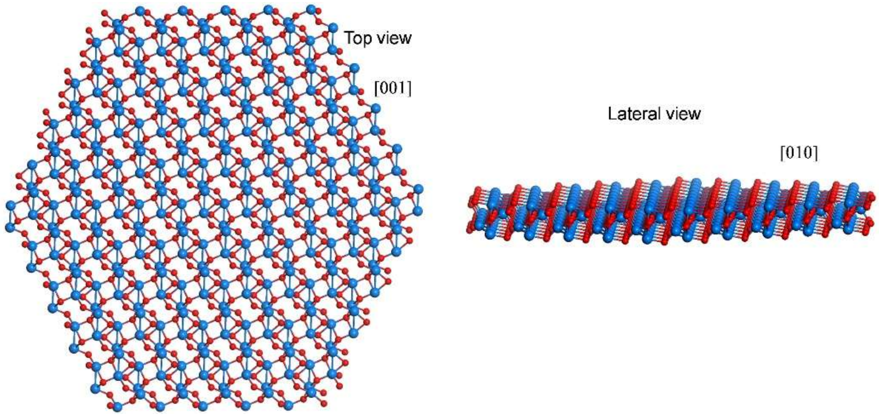

Fig. S3. Structural model of α-Fe_2O_3 nanosheets. The blue and red balls represent Fe and O atomic sites, respectively.

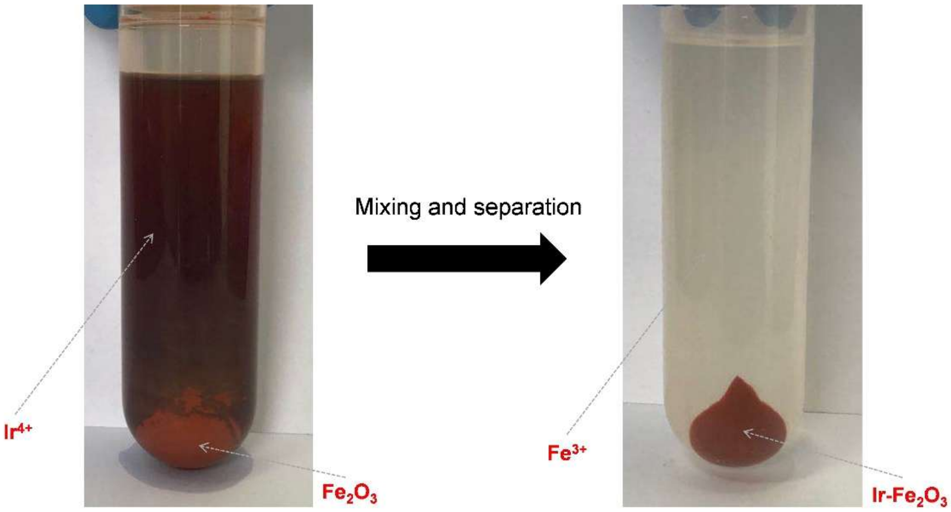

Fig. S4. Experimental verification of the adsorption of α-Fe_2O_3 nanosheets on Ir ions.

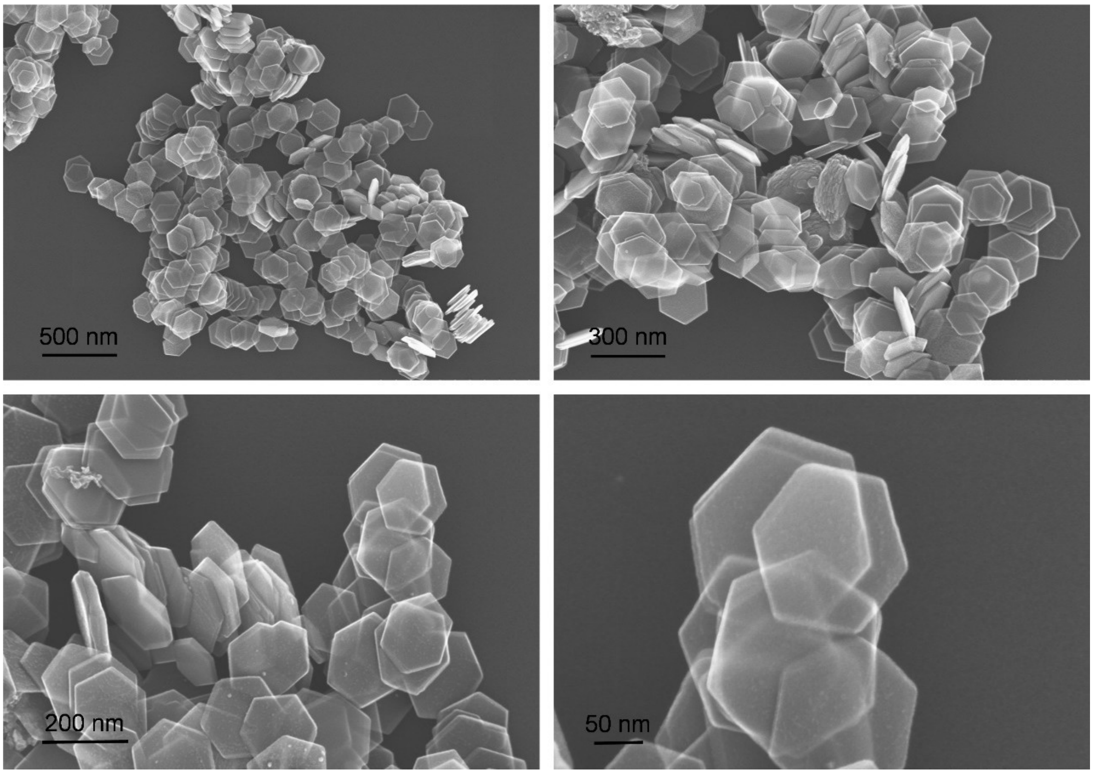

Fig. S5. SEM images of Ir-Fe_2O_3 nanosheets at different magnifications.

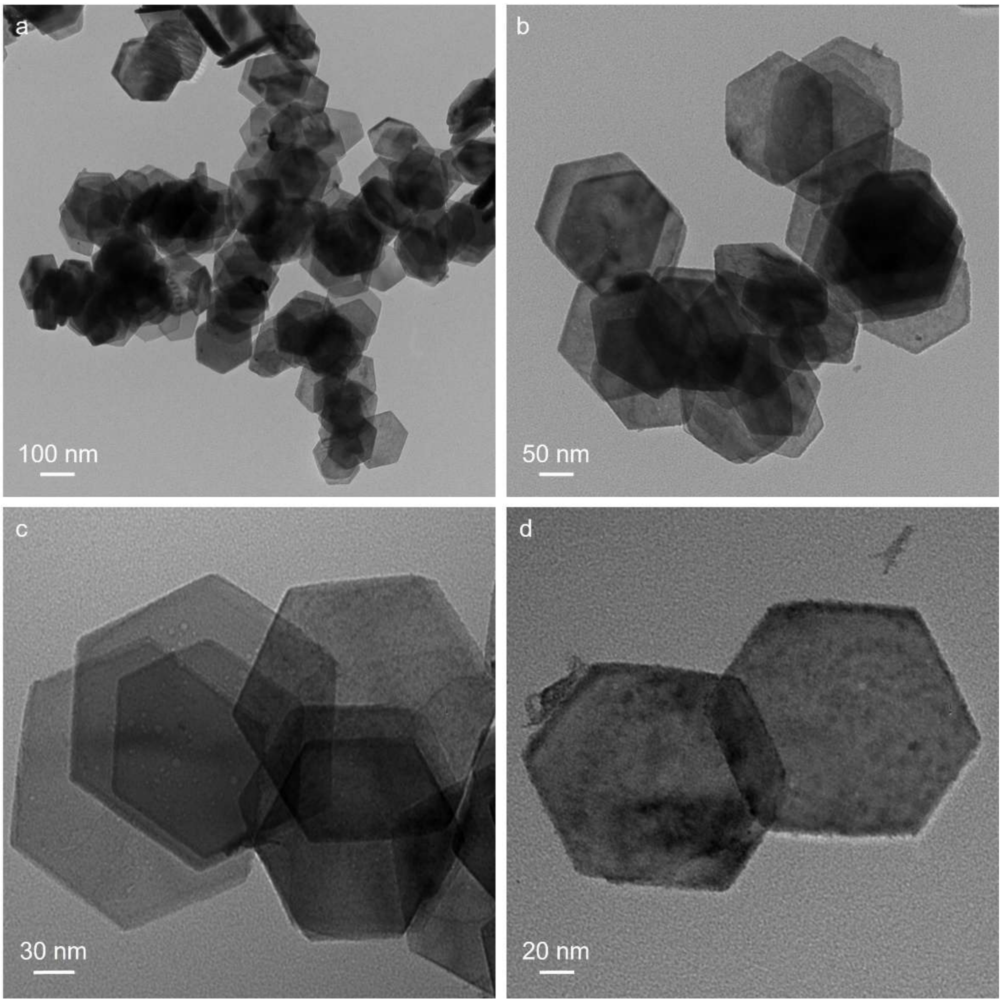

Fig. S6. (a-d) TEM images of Ir-Fe_2O_3 nanosheets at different magnifications.

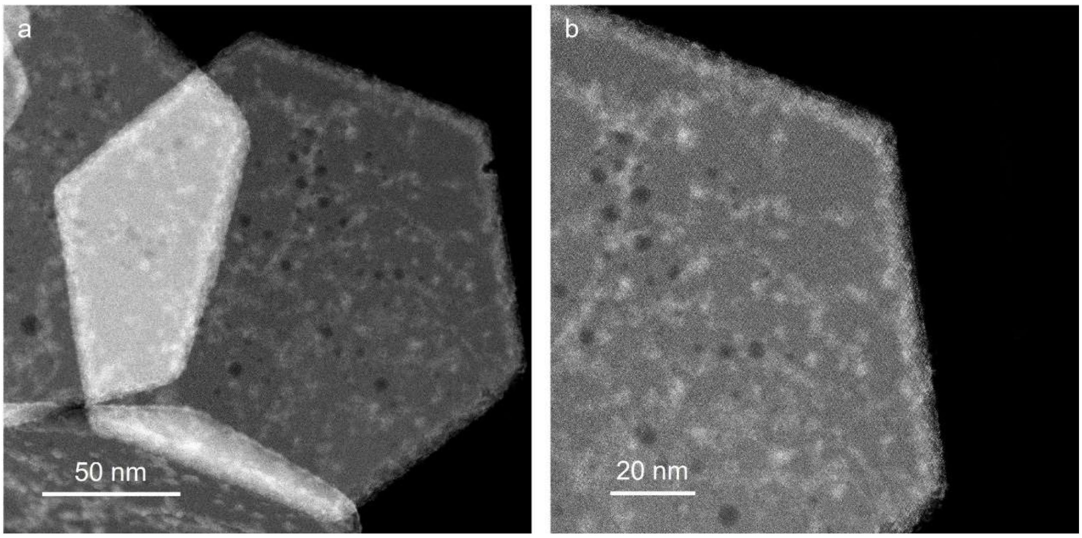

Fig. S7. (a, b) High-angle annular dark-field scanning TEM (HAADF-STEM) images of 12Ir-Fe_2O_3 at different magnifications.

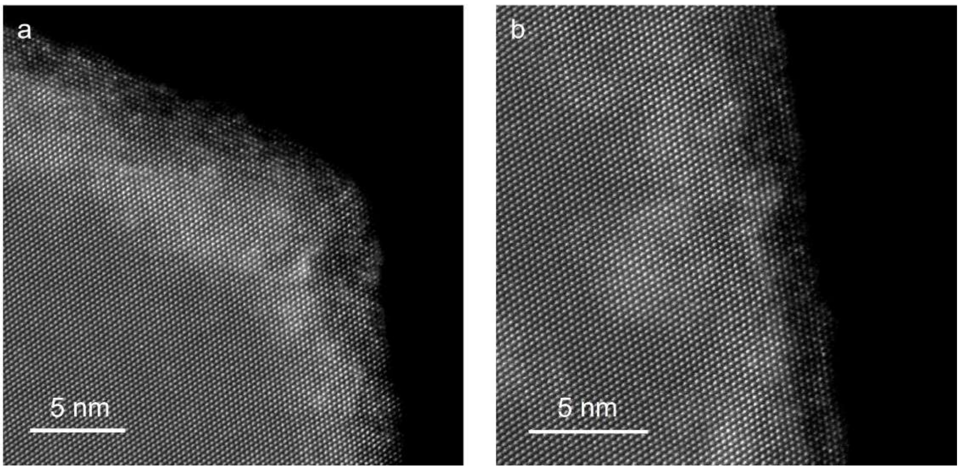

Fig. S8. (a, b) Atomic-resolution HAADF-STEM images of 12Ir-Fe_2O_3 in different selected areas.

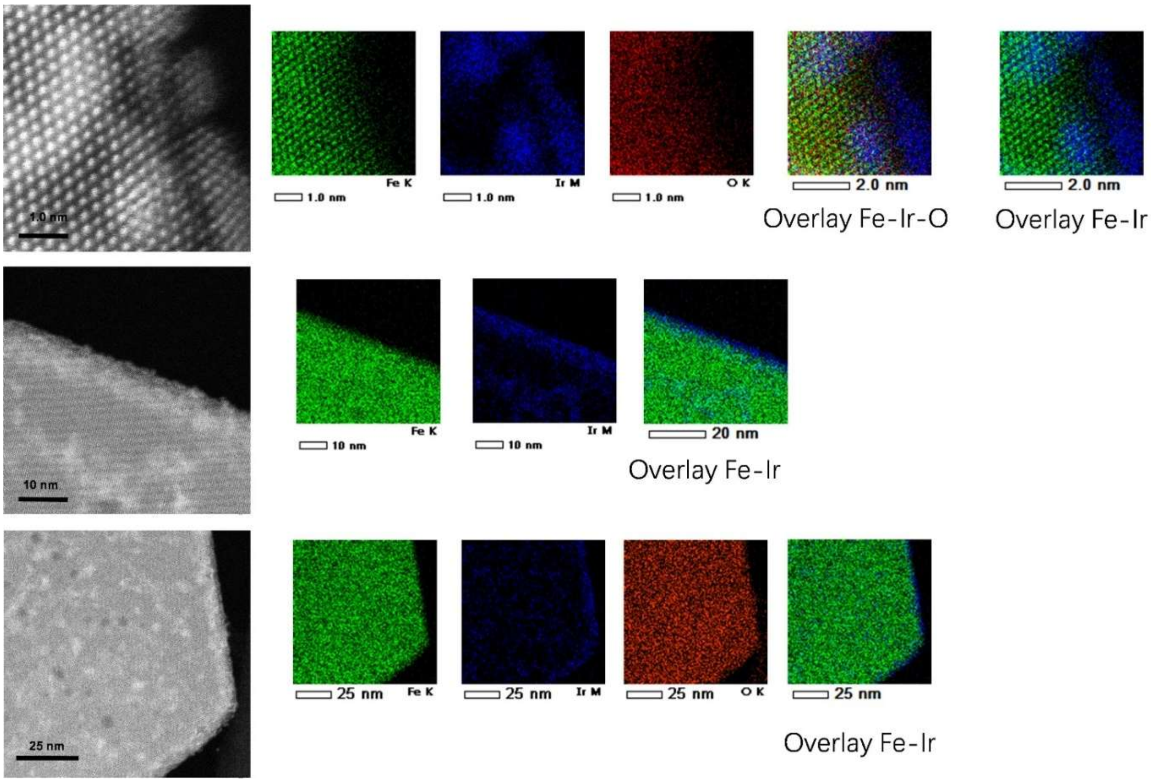

Fig. S9. Elemental mapping of 12Ir-Fe_2O_3 at different magnifications. It is worth noting that the atomic-resolution elemental mapping shown in **Fig. 2D** in the main text is taken from the upper part of this figure, corresponding to the atomic-resolution elemental mapping with a scale bar of 1 nm. Furthermore, the electron microscope image at the bottom of this figure, corresponding to a scale bar of 25 nm, is taken from **Fig. S7**.

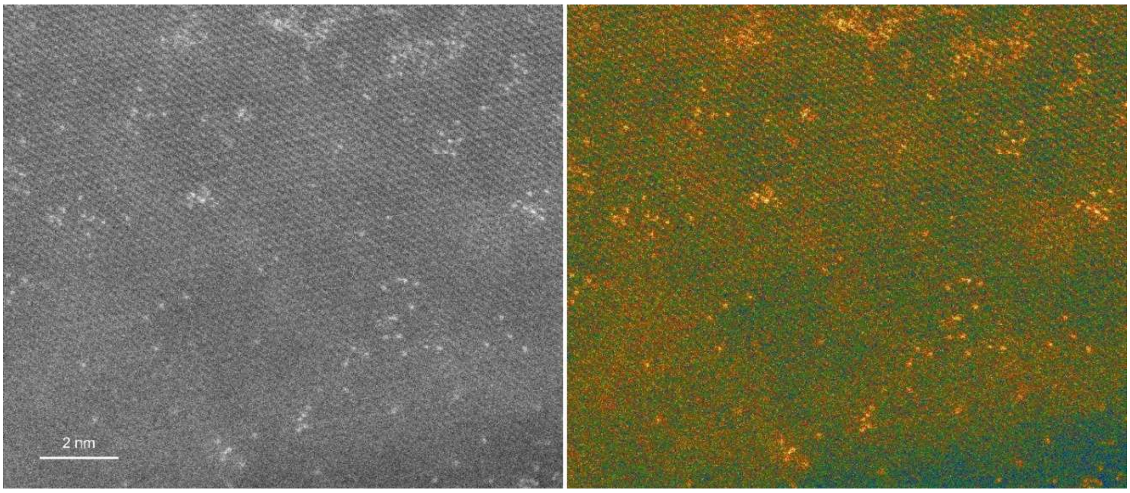

Fig. S10. HAADF-STEM images and corresponding color images of 2Ir-Fe_2O_3. Ir is mainly dispersed on the surface of Fe_2O_3 nanosheets in the form of single atoms.

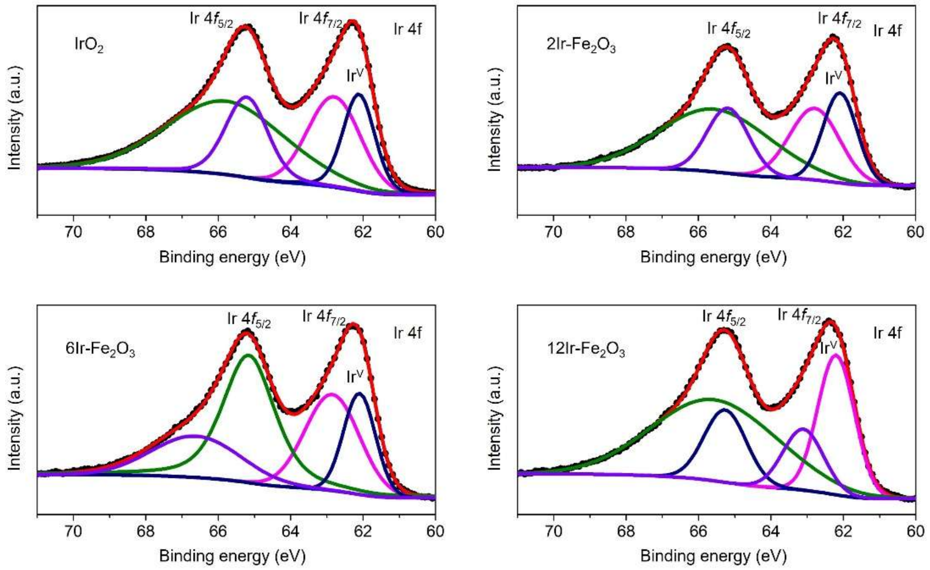

Fig. S11. Curve-resolved XPS of the Ir 4f region for IrO_2, 2Ir-Fe_2O_3, 6Ir-Fe_2O_3, and 12Ir-Fe_2O_3.

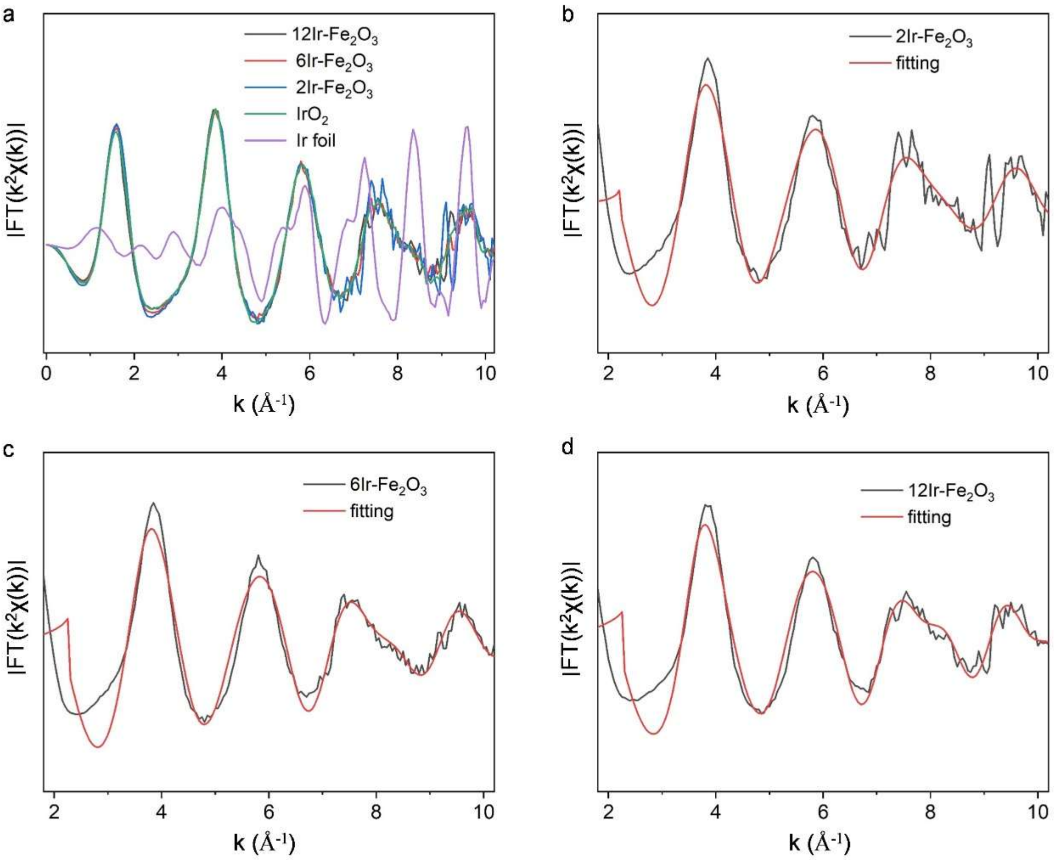

Fig. S12. Fourier transform of the EXAFS spectra at the L_3-edge of **(a)** a collection of all samples, **(b)** 2Ir-Fe_2O_3, **(c)** 6Ir-Fe_2O_3, and **(d)** 12Ir-Fe_2O_3 in k spaces. EXAFS fits for 2Ir-Fe_2O_3, 6Ir-Fe_2O_3, and 12Ir-Fe_2O_3 evidence a close match between the measured and calculated spectra. The fitted parameters are provided in **Table S1**.

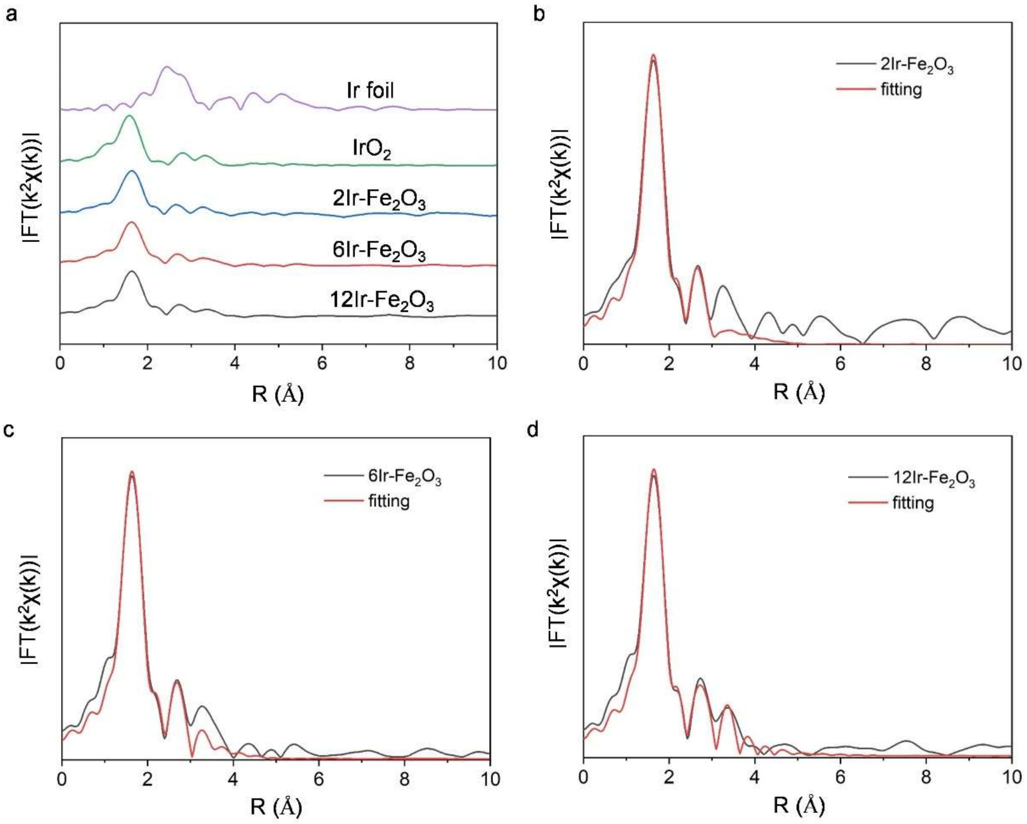

Fig. S13. Fourier transform of the EXAFS spectra at the L_3-edge of **(a)** a collection of all samples, **(b)** 2Ir-Fe_2O_3, **(c)** 6Ir-Fe_2O_3, and **(d)** 12Ir-Fe_2O_3 in R spaces.

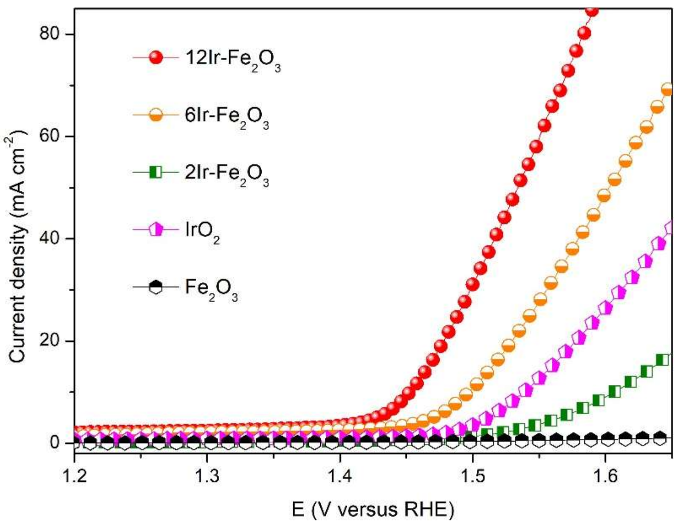

Fig. S14. OER polarization curves of xIr-Fe_2O_3 (x=2, 6, and 12), Fe_2O_3, and IrO_2 in 1 M $HClO_4$ without iR correction.

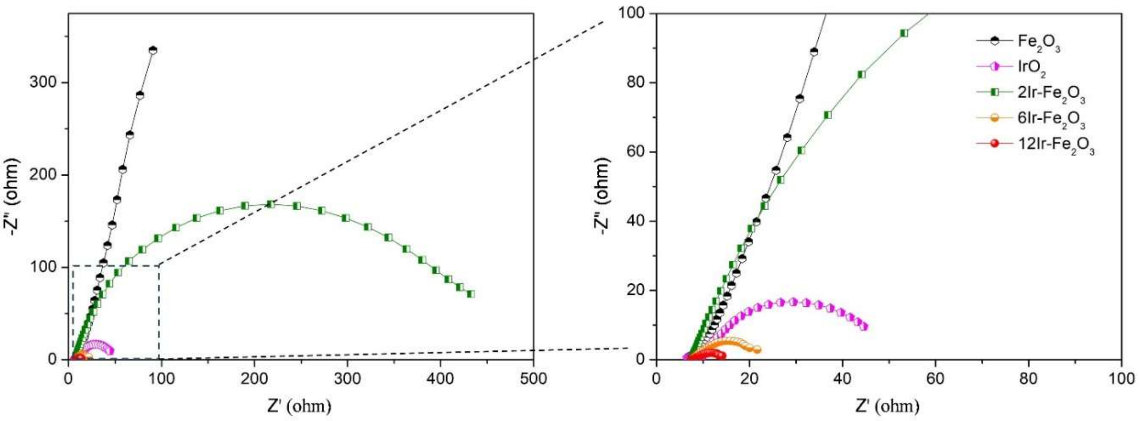

Fig. S15. Electrochemical impedance spectroscopy (EIS) plots of xIr-Fe_2O_3 (x=2, 6, and 12), IrO_2, and Fe_2O_3. X-axis (Z') is the real part, and Y-axis (-Z") is the imaginary part.

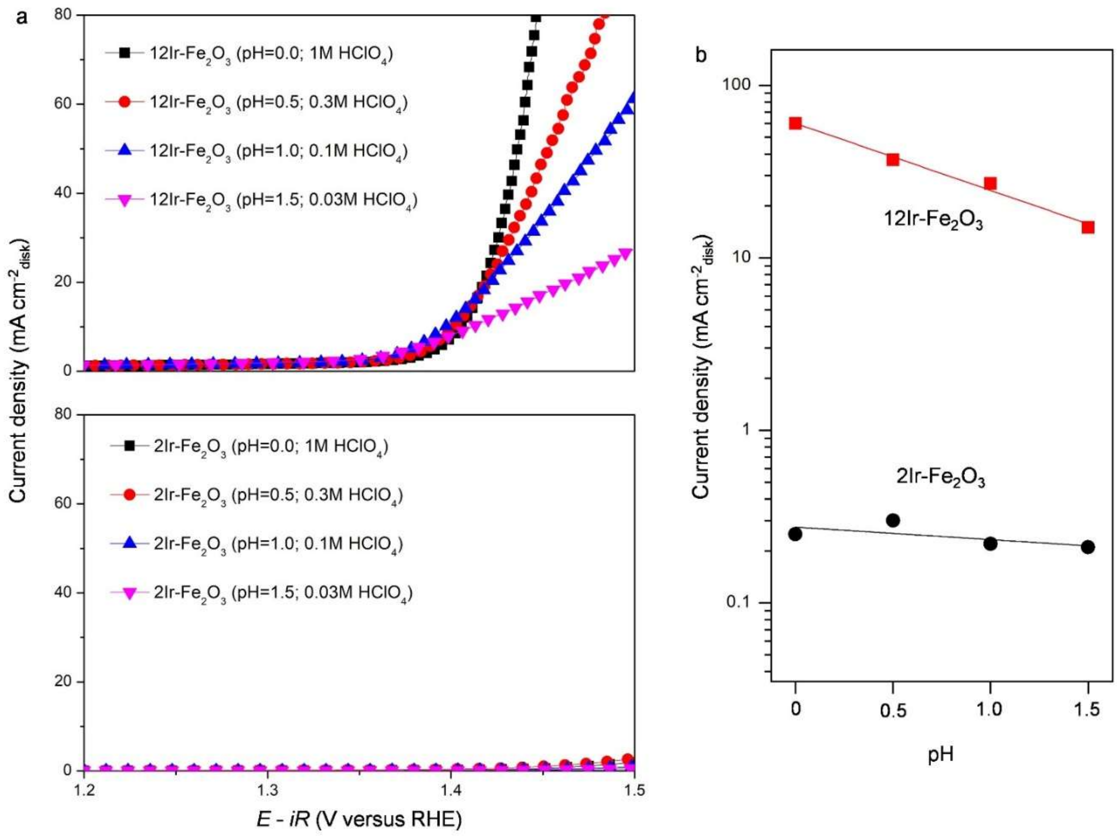

Fig. S16. (a) pH dependence of the OER activities of 12Ir-Fe_2O_3 and 2Ir-Fe_2O_3 catalysts. **(b)** Current densities of 12Ir-Fe_2O_3 and 2Ir-Fe_2O_3 at 1.43 V versus RHE as a function of the pH value.

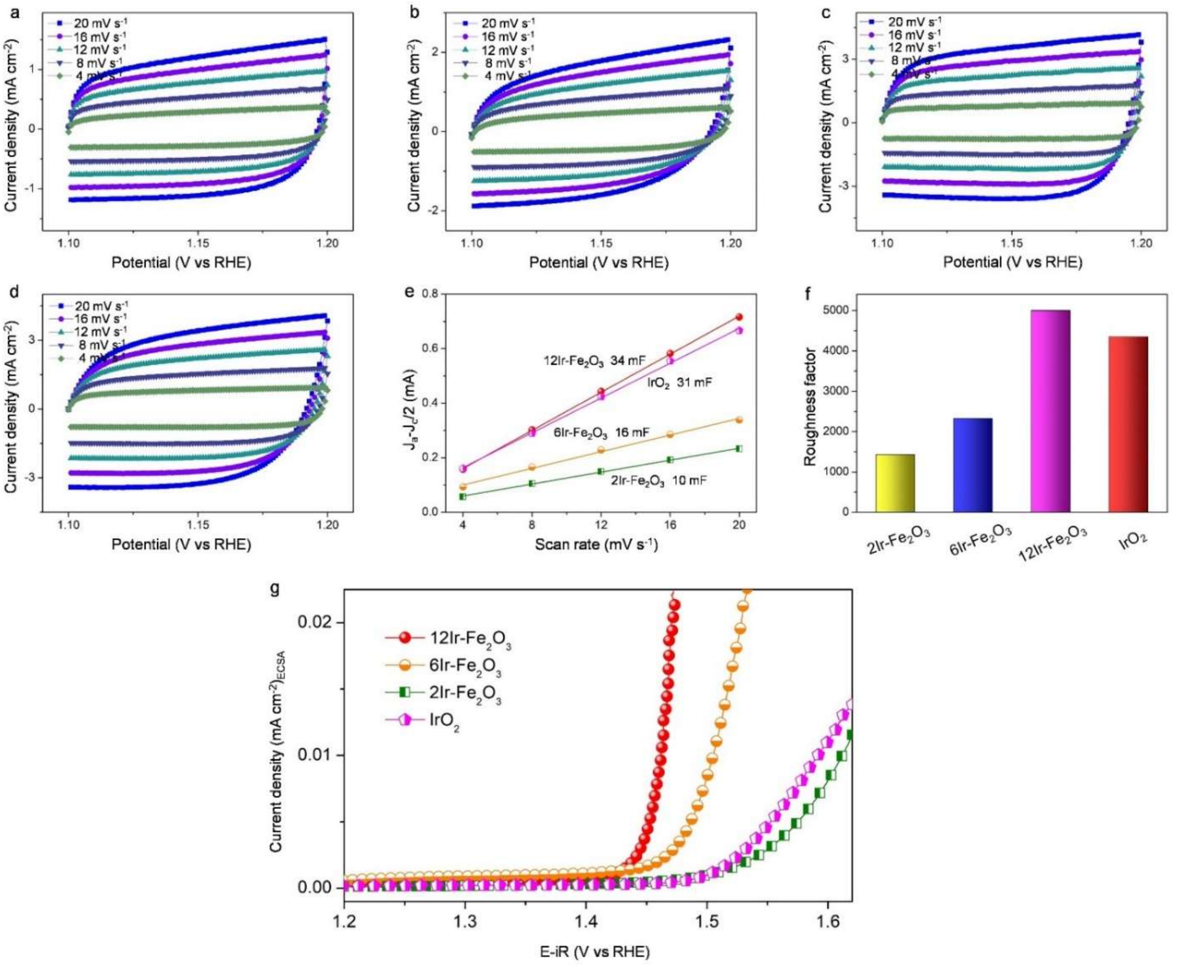

Fig. S17. CV profiles of **(a)** 2Ir-Fe_2O_3, **(b)** 6Ir-Fe_2O_3, **(c)** 12Ir-Fe_2O_3, and **(d)** IrO_2 catalysts in the non-Faradaic region of 1.1~1.2 V vs. RHE at the scan rates of 4 mV s^{-1}, 8 mV s^{-1}, 12 mV s^{-1}, 16 mV s^{-1}, and 20 mV s^{-1}. **(e)** C_{dl} plots obtained from CV curves. **(f)** Roughness factors for different catalysts (see *Method*). **(g)** Normalized LSV curves to electrochemically active surface area.

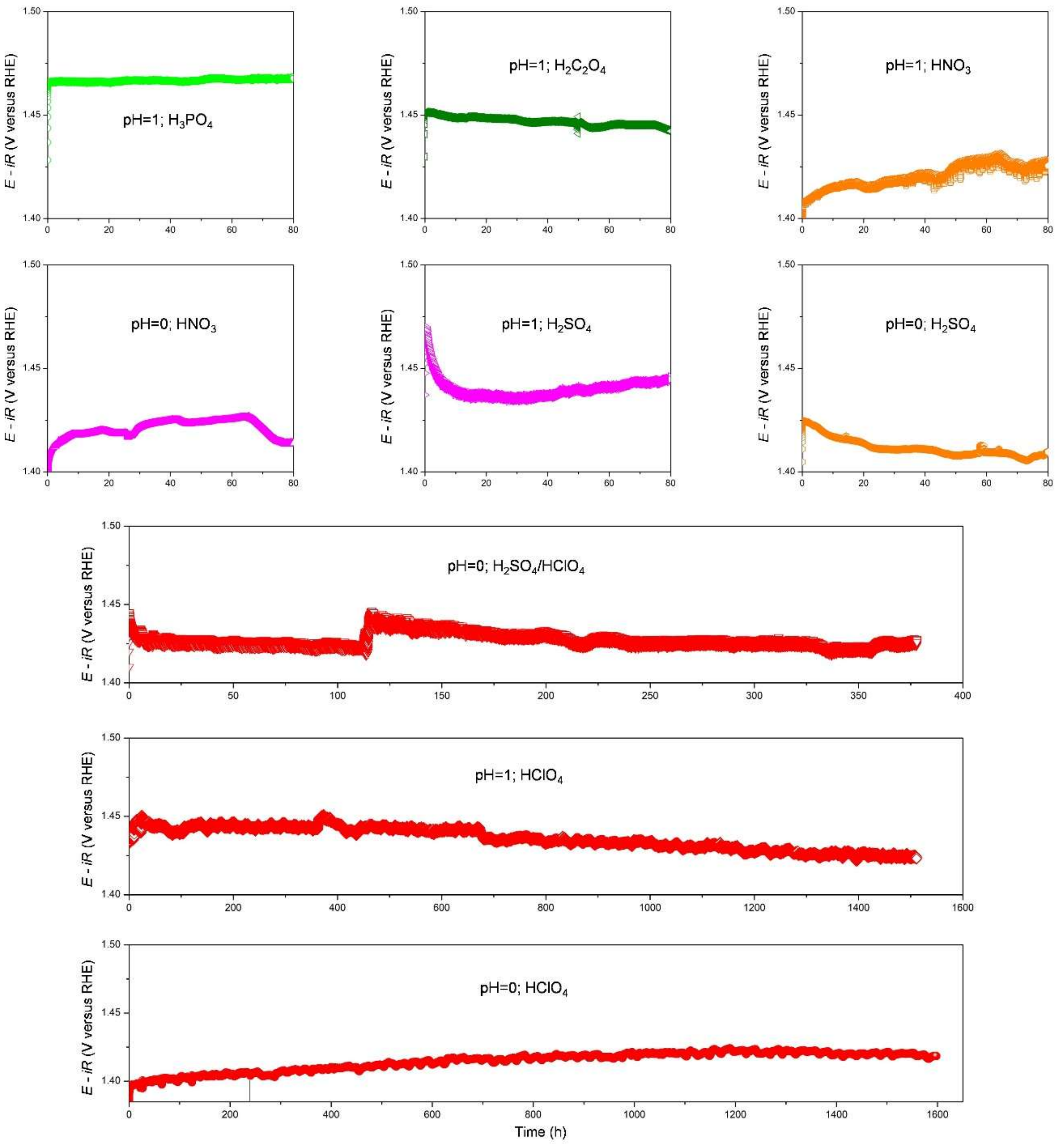

Fig. S18. Time dependence of the electrochemical potential necessary to perform OER at 10 mA cm^{-2} in H_3PO_4 (pH = 1), $H_2C_2O_4$ (pH = 1), HNO_3 (pH = 1), HNO_3 (pH = 0), H_2SO_4 (pH = 1), H_2SO_4 (pH = 0), $H_2SO_4/HClO_4$ (pH = 0), $HClO_4$ (pH = 1) and $HClO_4$ (pH = 0), respectively. A narrower y-axis range was used to enhance the visibility of variations in these stability plots.

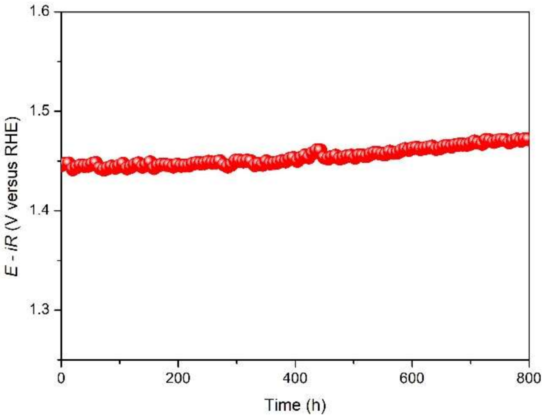

Fig. S19. Time dependence of the electrochemical potential necessary to perform OER at 10 mA cm^{-2} in H_2SO_4.

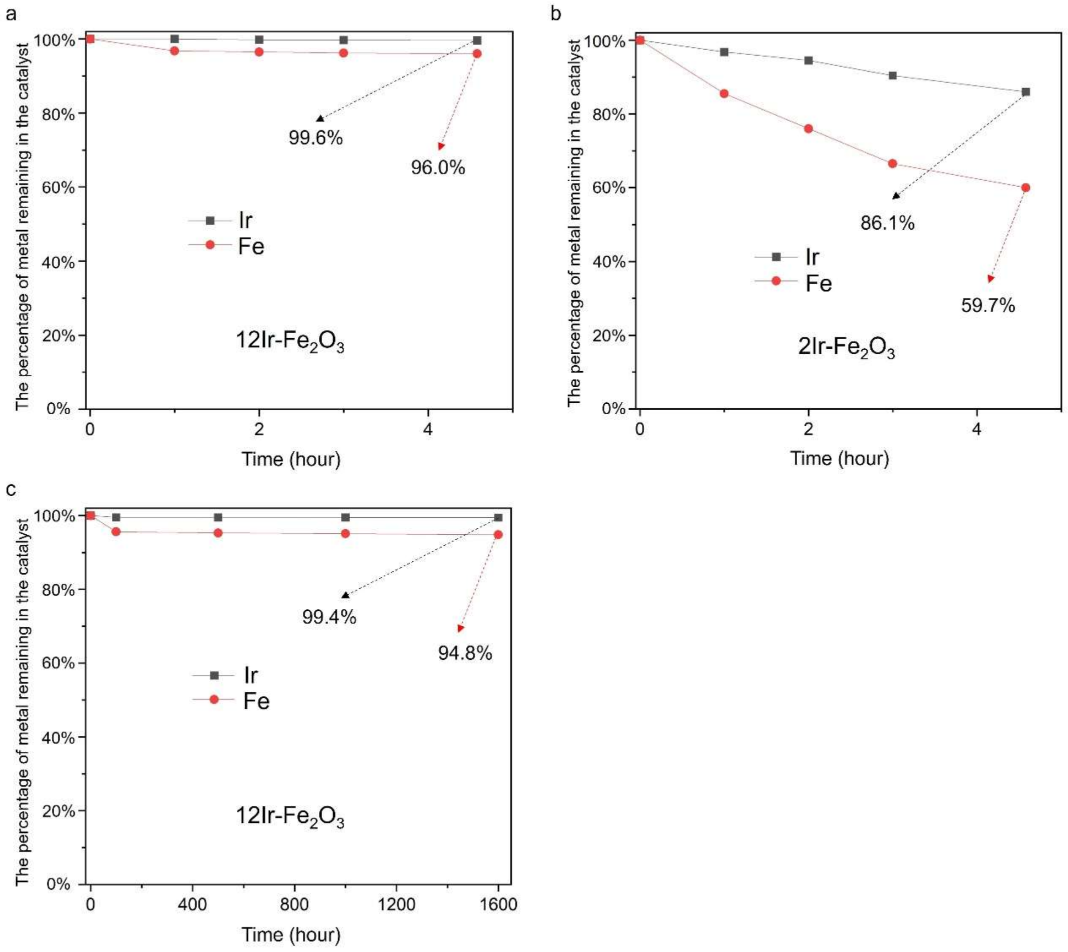

Fig. S20. Dependence of the residual percentage of Ir and Fe in the catalyst on the reaction time during the OER process, using (**a**, **c**) 12Ir-Fe_2O_3 and (**b**) 2Ir-Fe_2O_3 as working electrodes, respectively. The OER current density was set to 10 mA cm^{-2} and the electrolyte was $HClO_4$ at pH = 0.

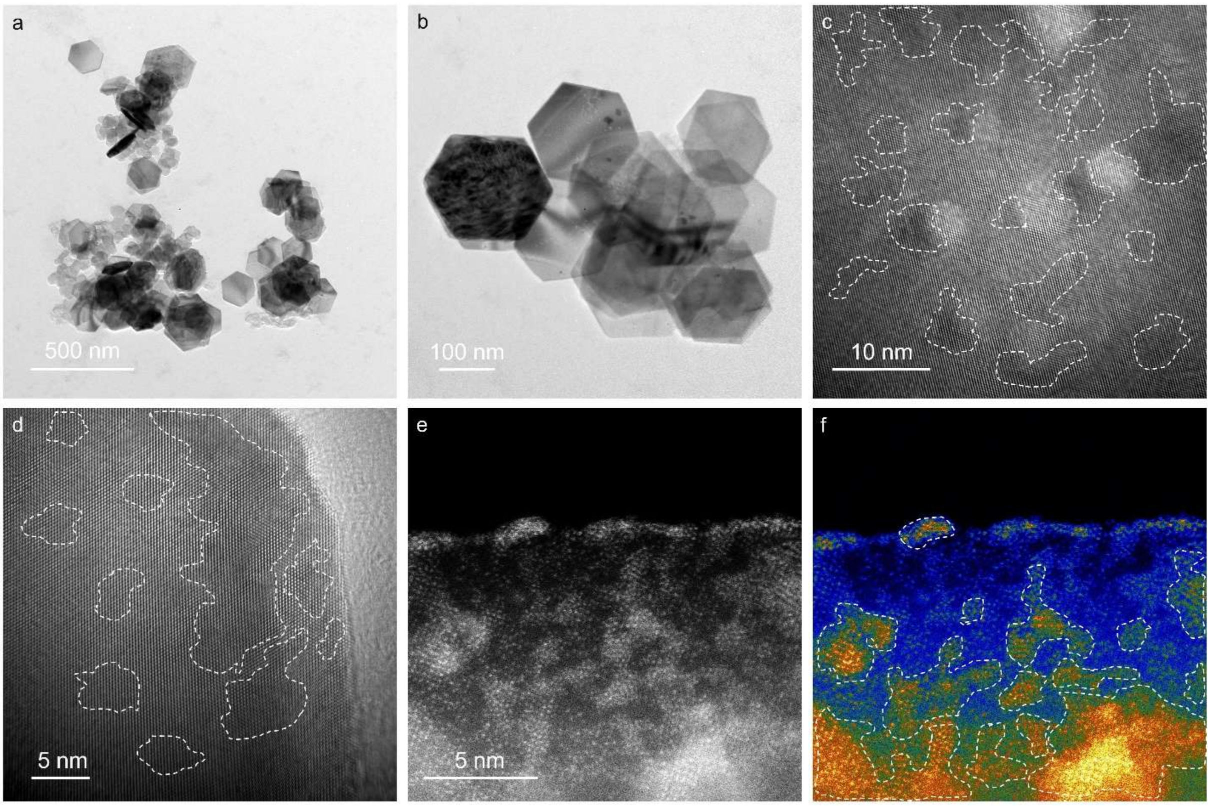

Fig. S21. (a-d) TEM images of the 12Ir-Fe_2O_3 catalyst after the long-term OER stability test at different magnifications. **(e, f)** HAADF-STEM images and corresponding color images of the 12Ir-Fe_2O_3 catalyst after the long-term stability test.

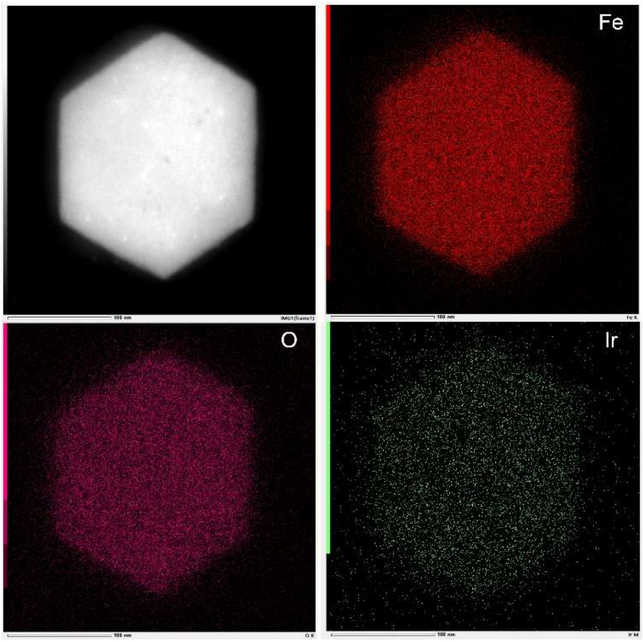

Fig. S22. STEM image of the spent $12Ir-Fe_2O_3$ catalyst and the corresponding EDS element mapping images.

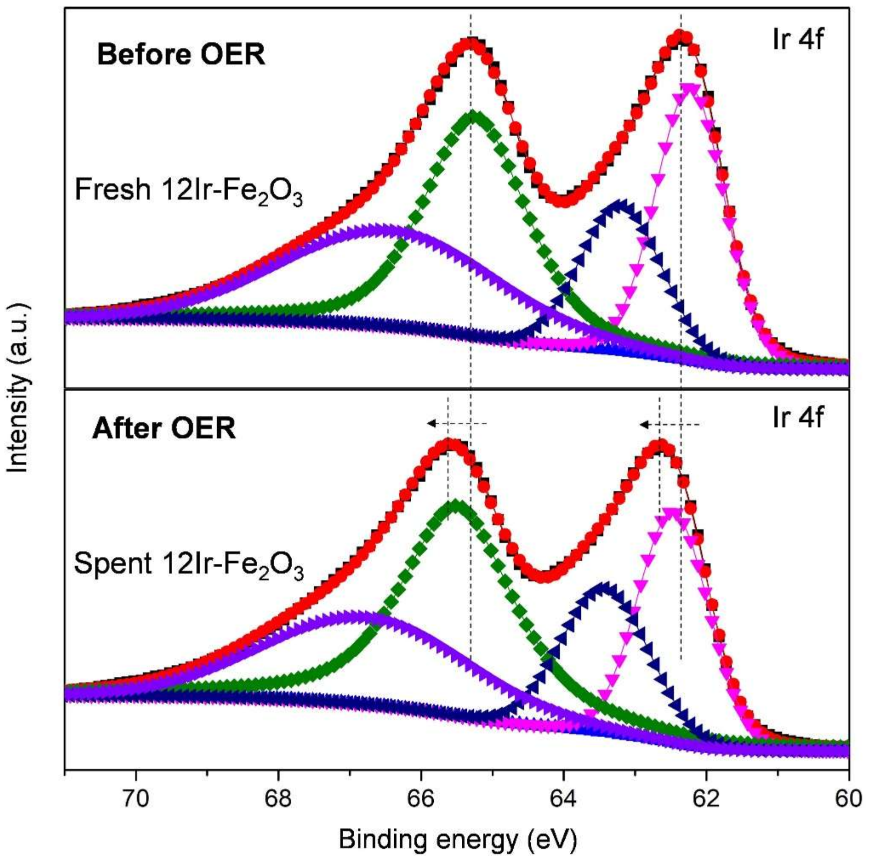

Fig. S23. High-resolution XPS Ir 4f of fresh and spent 12Ir-Fe_2O_3. The peak position of Ir 4f in the spent 12Ir-Fe_2O_3 is slightly shifted to a higher binding energy because of the oxidative potential.

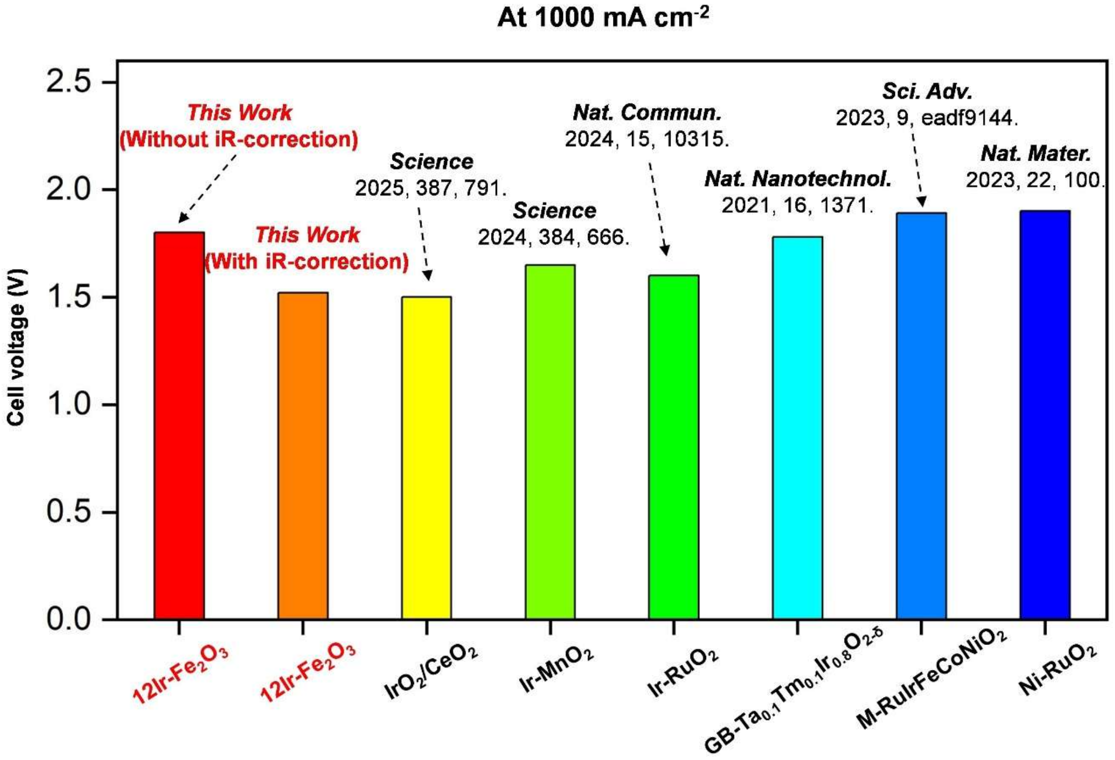

Fig. S24. Cell voltages corresponding to different OER catalysts reported in recent literature at a current density of 1000 mA cm^{-2} in PEM electrolyzer.

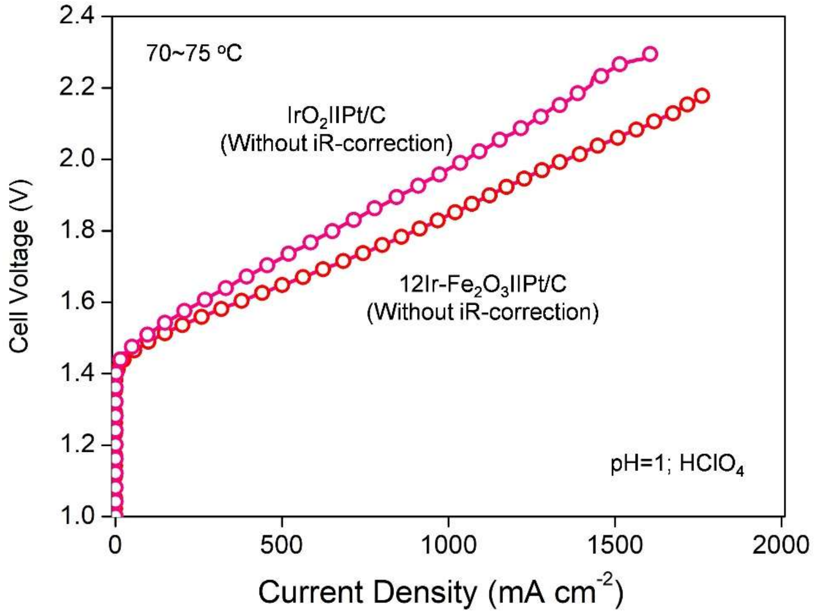

Fig. S25. Polarization curves of the PEM electrolysers using 12Ir-Fe_2O_3 and IrO_2 as anode catalysts.

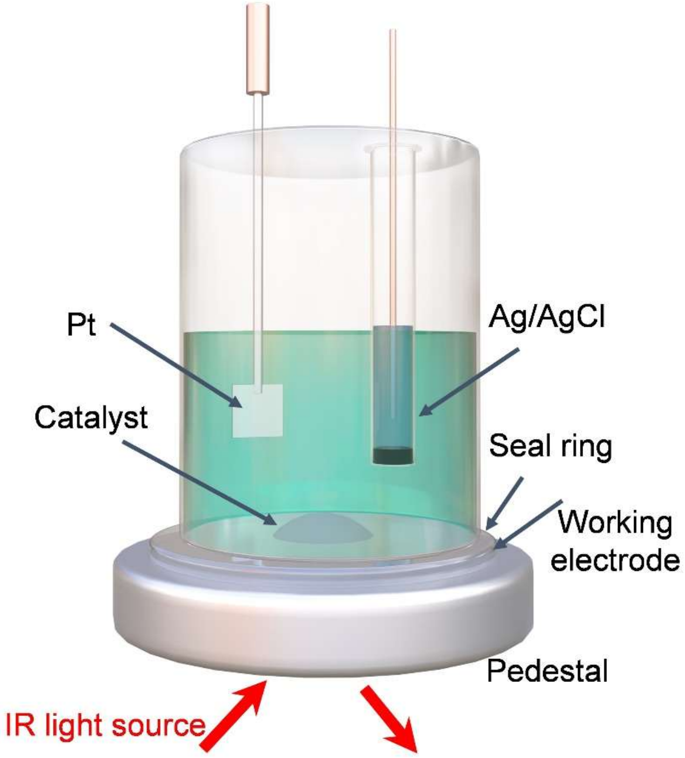

Fig. S26. Schematic diagram of the in-situ IR experimental setup.

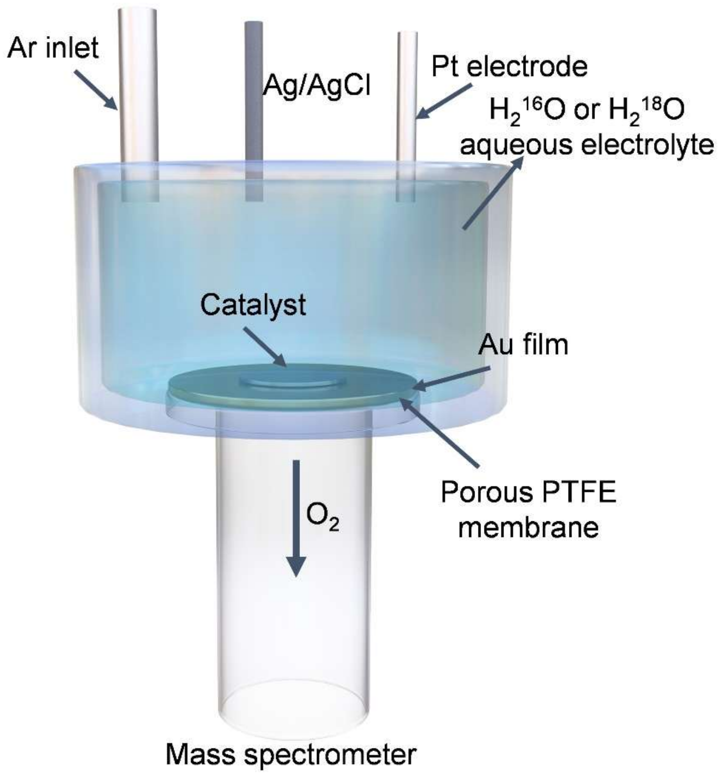

Fig. S27. Schematic diagram of the in-situ DEMS experimental setup.

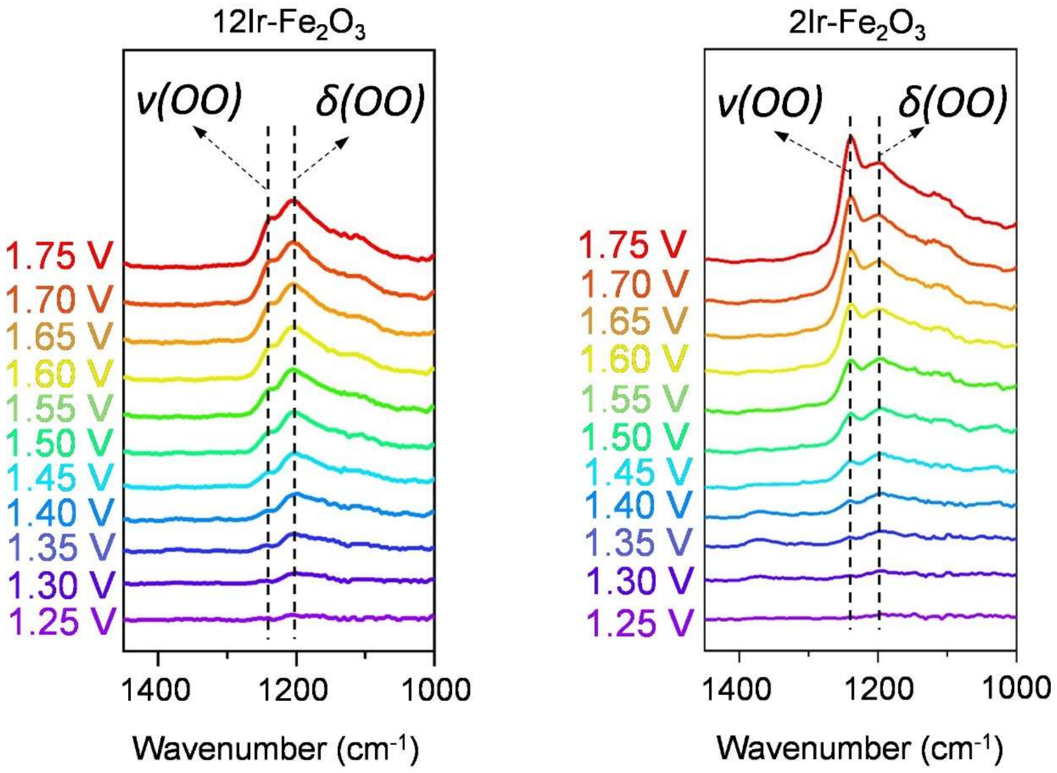

Fig. S28. In-situ ATR-SEIRAS measurements in the range of 1000-1450 cm^{-1} under various potentials for 12Ir-Fe_2O_3 catalyst and 2Ir-Fe_2O_3 catalyst during the OER process.

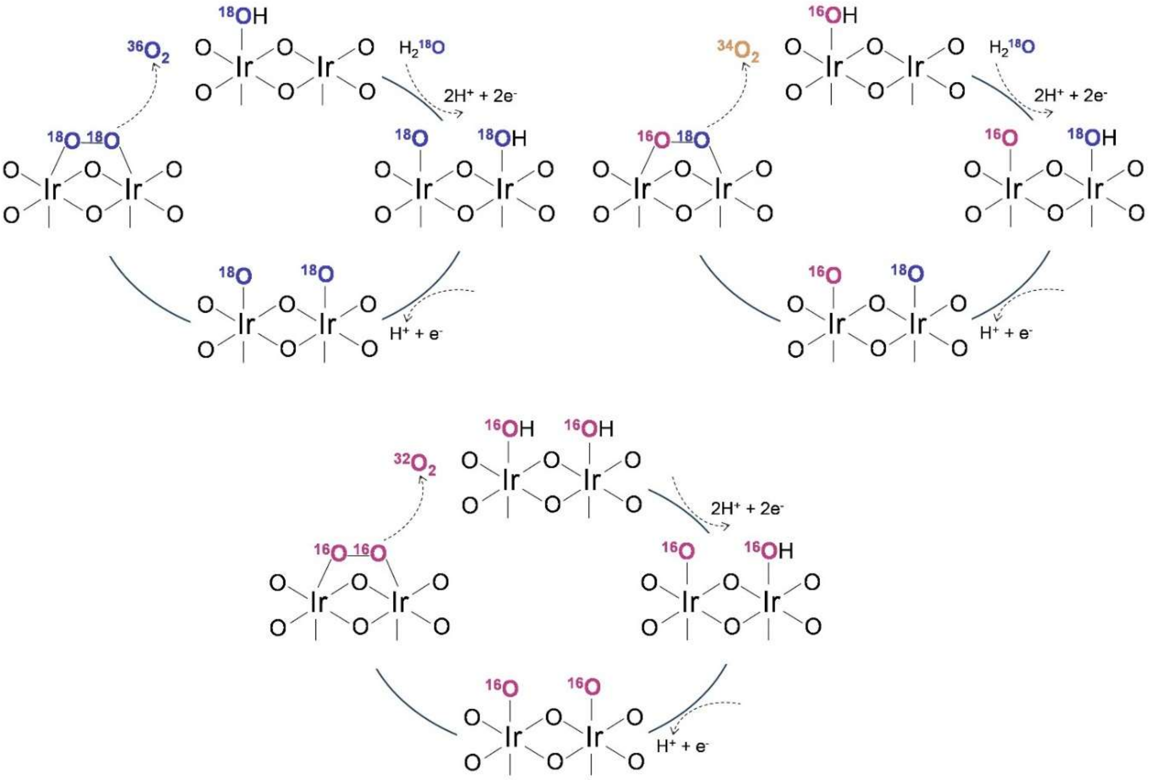

Fig. S29. The speculated mechanism for different isotope-labeled O_2 products in 12Ir-Fe_2O_3 involves a dual-metal site process. The evolution pathways of $^{32}O_2$ ($^{16}O^{16}O$), $^{34}O_2$ ($^{18}O^{16}O$), and $^{36}O_2$ ($^{18}O^{18}O$) in the electrolyte, using $H_2^{18}O$ as the solvent, were examined. In the first two pathways, $H_2^{18}O$ participates in the OER. In contrast, the third pathway involves only surface adsorbates containing ^{16}O in the OER.

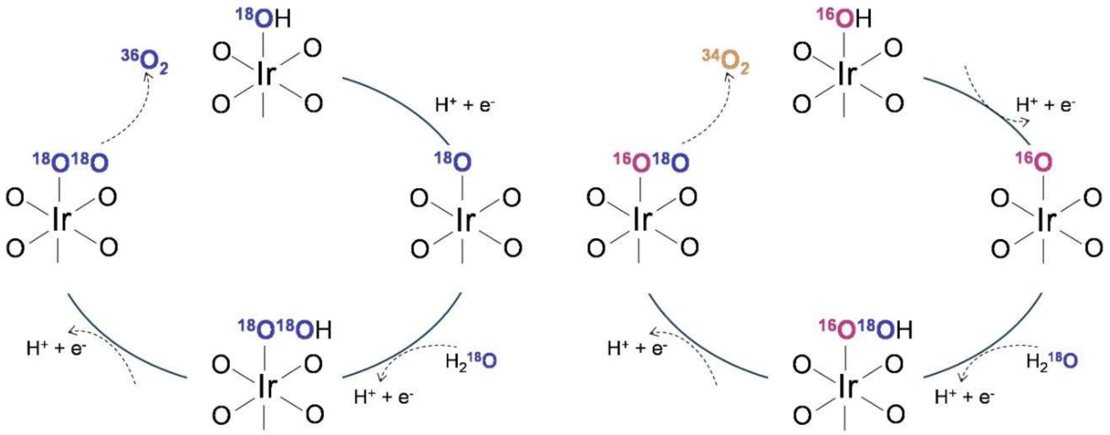

Fig. S30. The speculated mechanism for different isotope-labeled O_2 products involves a single-metal site process. The evolution pathways of $^{36}O_2$ ($^{18}O^{18}O$) and $^{34}O_2$ ($^{18}O^{16}O$) in the electrolyte, using $H_2{}^{18}O$ as the solvent, were analyzed.

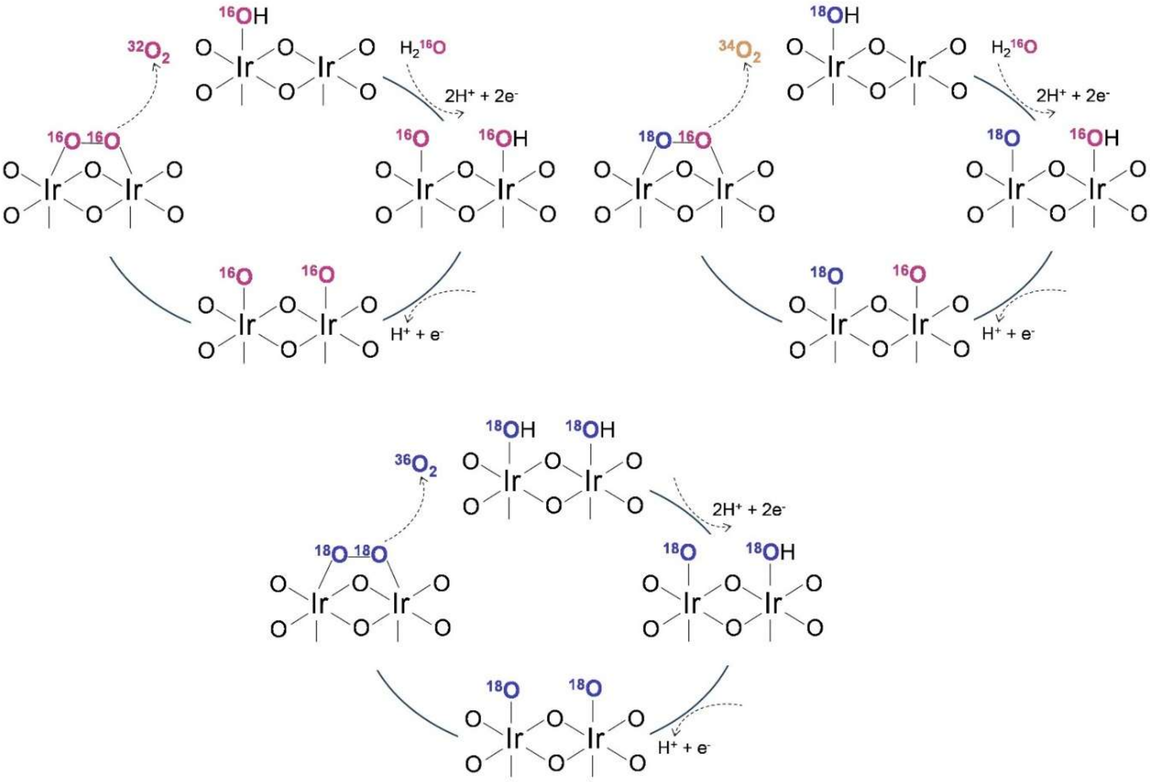

Fig. S31. The speculated mechanism for different isotope-labeled O_2 products in 12Ir-Fe_2O_3 follows a dual-metal site process. The evolution pathways of $^{32}O_2$ ($^{16}O^{16}O$), $^{34}O_2$ ($^{18}O^{16}O$), and $^{36}O_2$ ($^{18}O^{18}O$) in the electrolyte, using $H_2{}^{16}O$ as the solvent, were analyzed. In the first two pathways, $H_2{}^{16}O$ participates in the OER, while the third pathway involves only surface adsorbates containing ^{18}O in the OER.

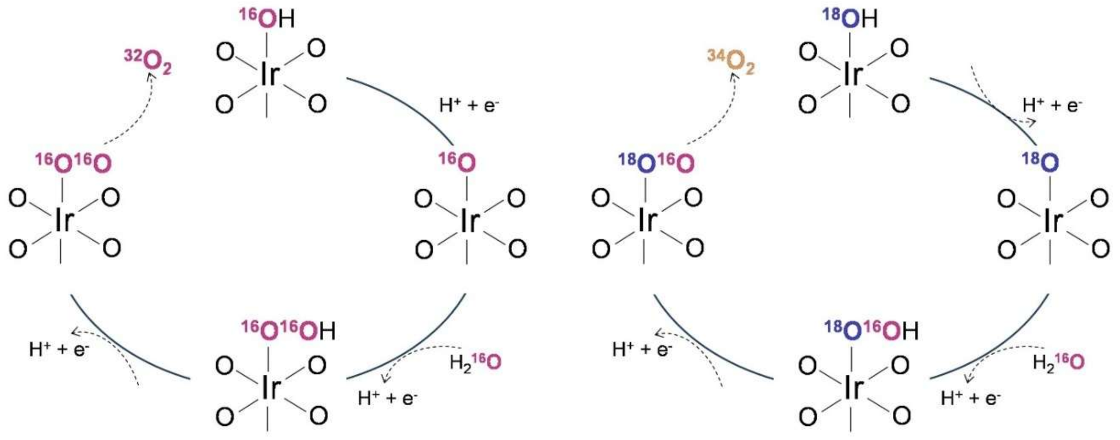

Fig. S32. The speculated mechanism for different isotope-labeled O_2 products follows a single-metal site process. The evolution pathways of $^{32}O_2$ ($^{16}O^{16}O$) and $^{34}O_2$ ($^{18}O^{16}O$) in the electrolyte, using $H_2{}^{16}O$ as the solvent, were analyzed.

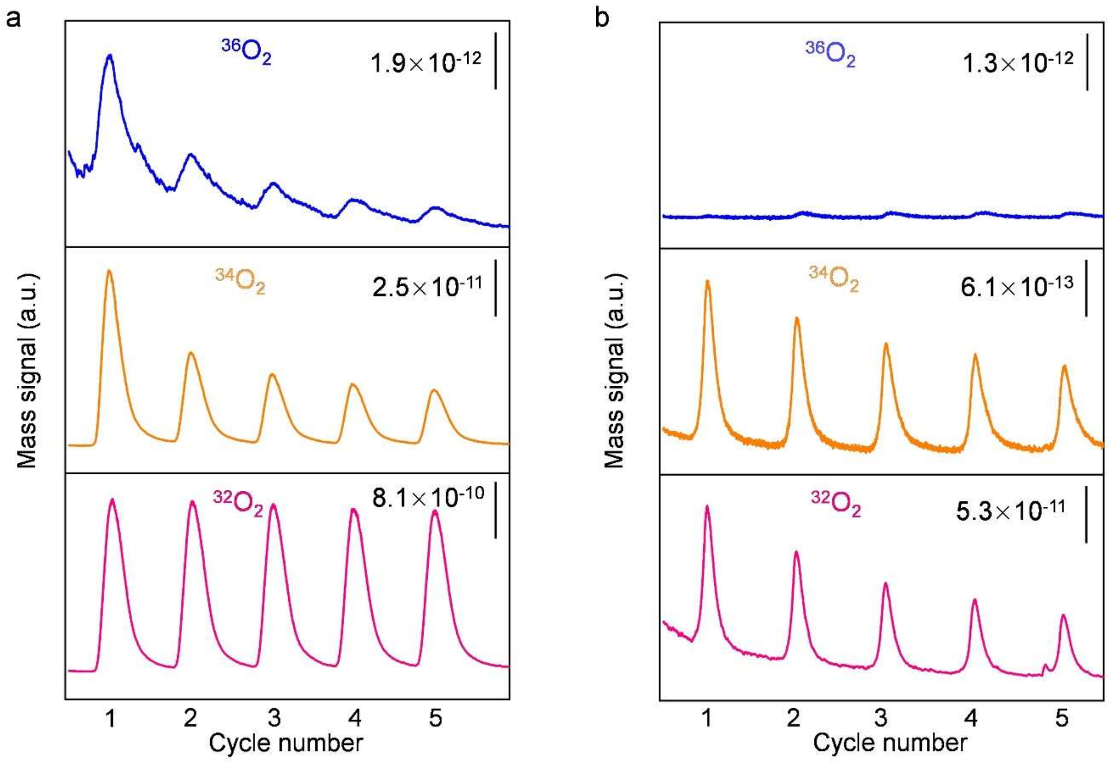

Fig. S33. Operando DEMS with the isotope labeling measurements. DEMS signals of O_2 products for **(a)** ^{18}O-surface labeled 12Ir-Fe_2O_3, and **(b)** ^{18}O-surface labeled 2Ir-Fe_2O_3 in the electrolyte using $H_2{}^{16}O$ as solvent. The black vertical lines on the top of the spectra (**a** and **b**) show the scale of mass signal.

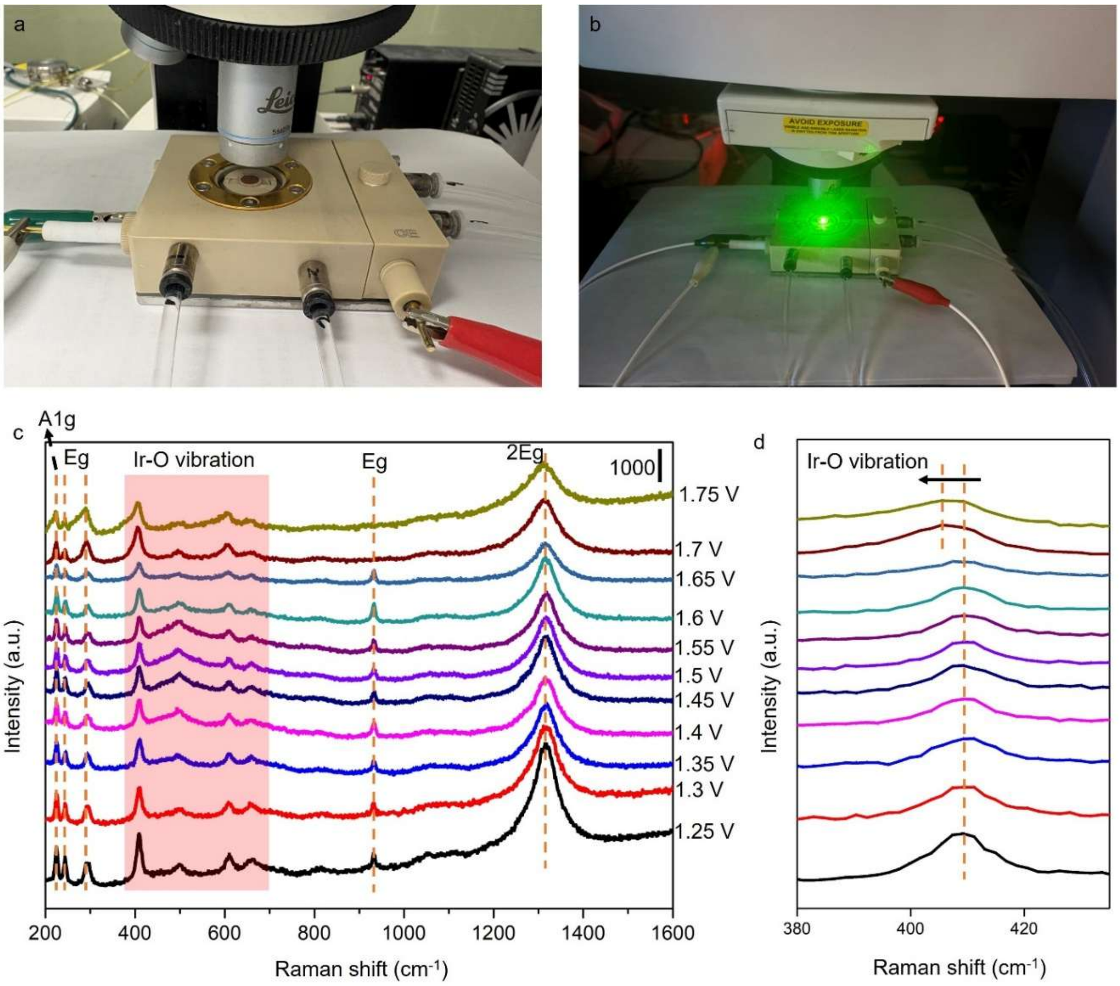

Fig. S34. (a, b) Image of in-situ Raman experimental setup. **(c, d)** In-situ Raman spectra of 12Ir-Fe_2O_3 catalyst at different applied potentials and their magnified images. One A1g mode, three Eg modes, and one 2Eg mode marked in (**c**) are characteristic vibrational peaks of Fe_2O_3. With increasing applied potential, the Eg peak near 930 cm^{-1} gradually weakens and eventually disappears, which is likely due to intensified gas bubble generation at high current densities that partially masks the Raman signal.

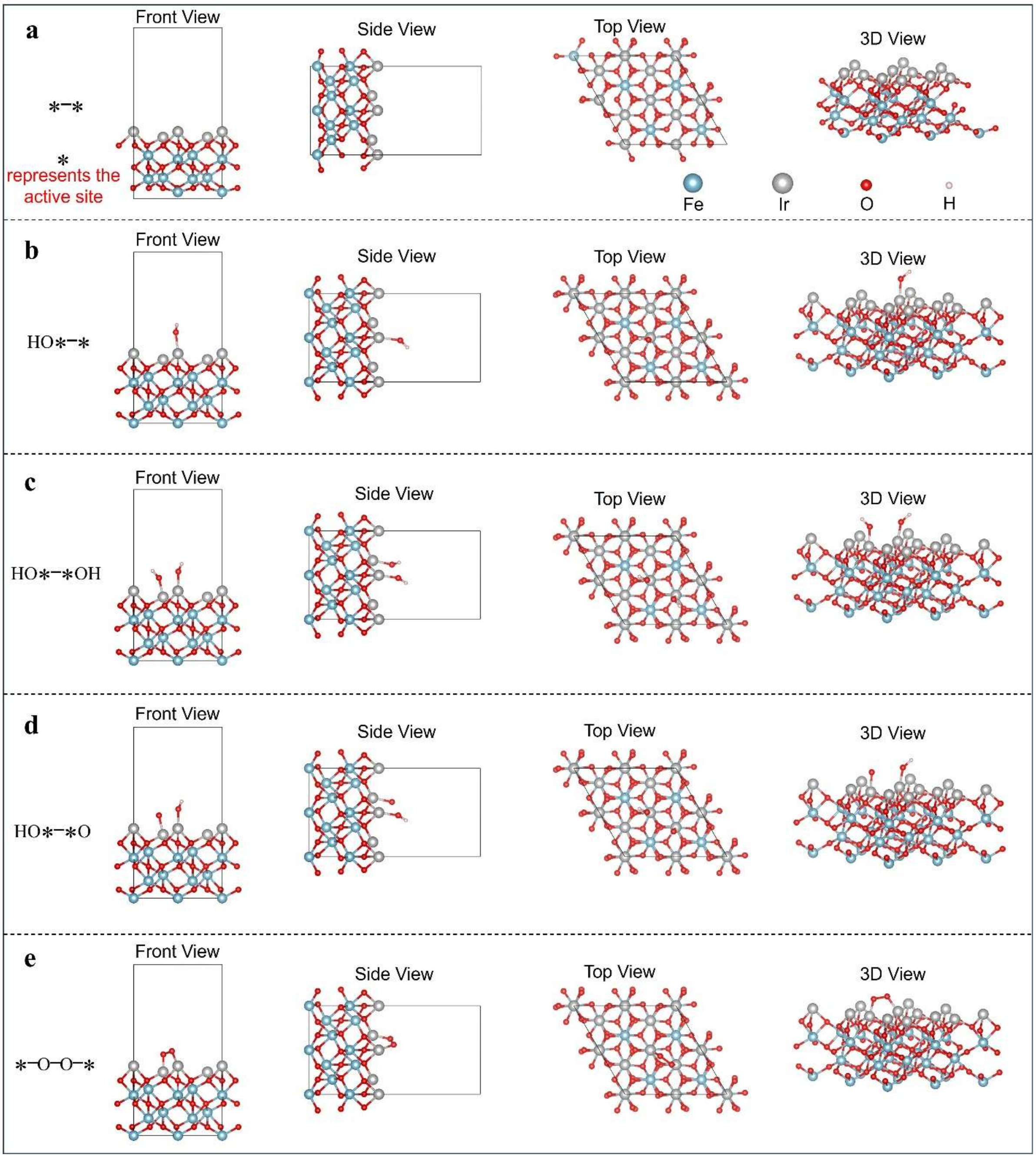

Fig. S35. Structural models for different states during oxygen evolution on Ir atom patches based on the dual-active-site mechanism. (**a**) Initial OER state with dual Ir active sites, shown from four viewing angles. (**b**) OH adsorption on one Ir site, shown from four viewing angles. (**c**) OH adsorption on both Ir sites, shown from four viewing angles. (**d**) Co-adsorption of OH and O on the two Ir sites, shown from four viewing angles. (**e**) O–O adsorption on the dual Ir sites, shown from four viewing angles.

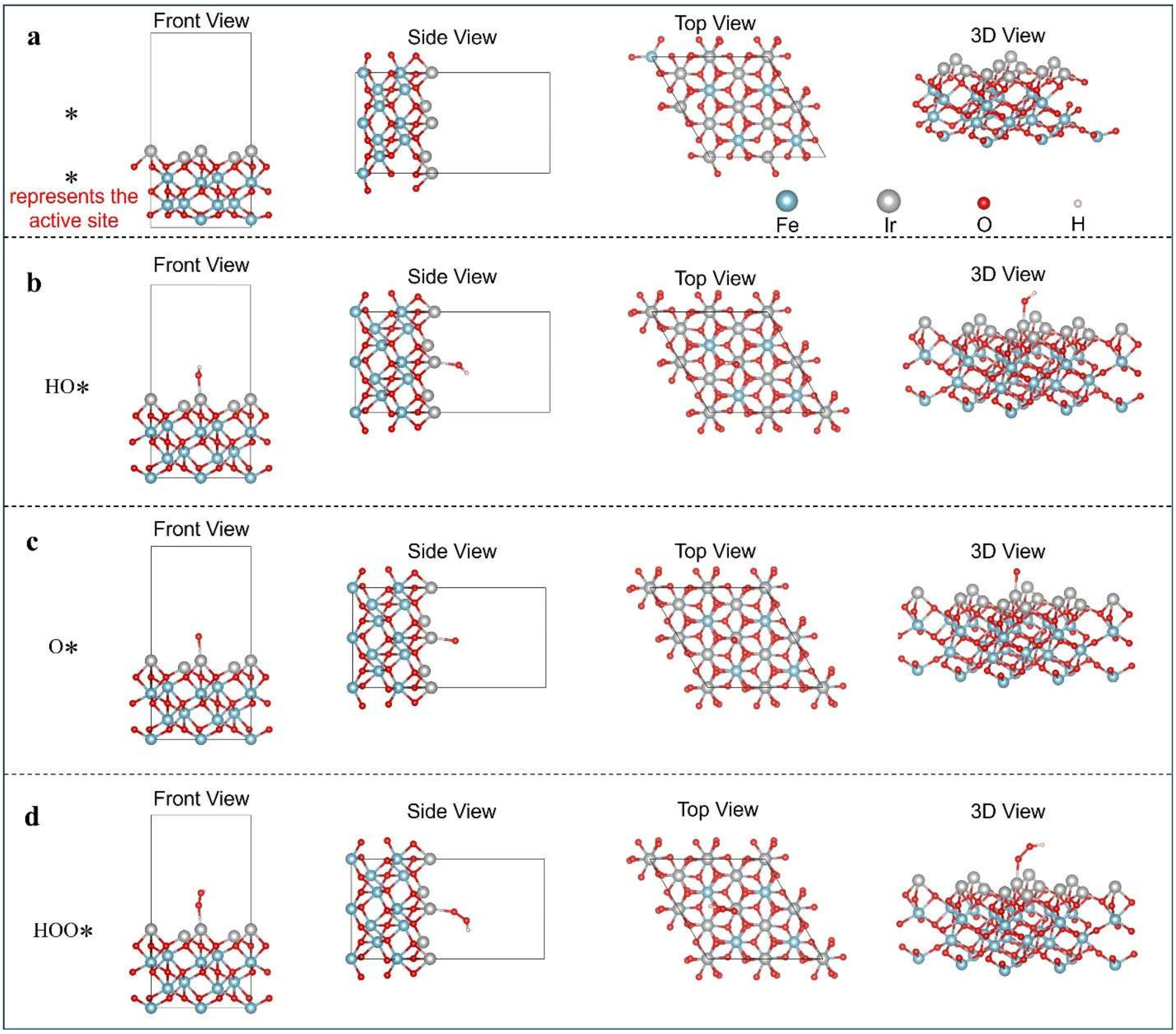

Fig. S36. Structural models for different states during oxygen evolution on Ir atom patches based on the single-metal site mechanism. (**a**) Initial OER state with single Ir active sites, shown from four viewing angles. (**b**) OH adsorption on one Ir site, shown from four viewing angles. (**c**) O adsorption on one Ir sites, shown from four viewing angles. (**d**) OOH adsorption on the one Ir site, shown from four viewing angles.

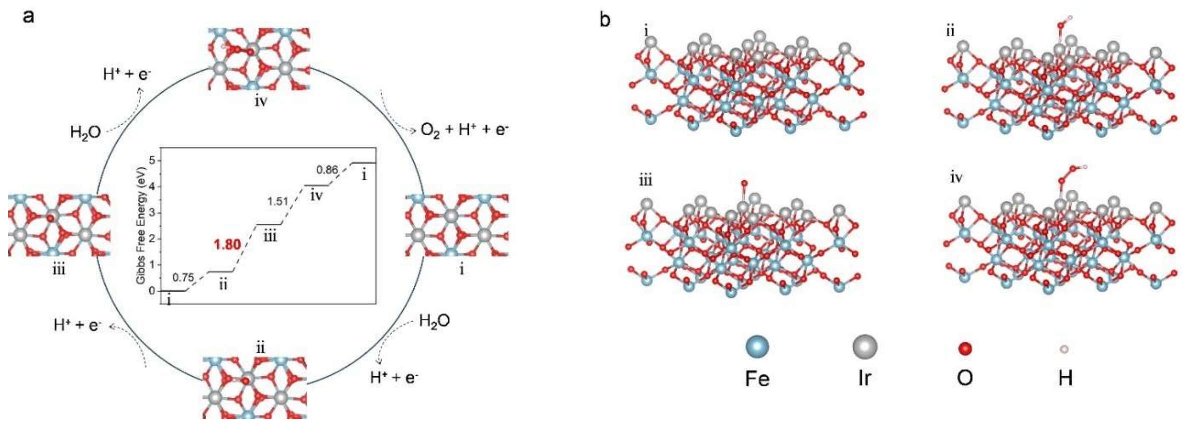

Fig. S37. (a) Top view and **(b)** side view of the OER reaction pathway based on the single-metal site mechanism on Ir atomic patch catalyst. The inset in (**a**) shows the calculated energy profile, where the initial Ir atomic patch system is used as the reference for the energy (in eV). After one catalytic cycle, the catalyst is recycled and releases one O_2 molecule.

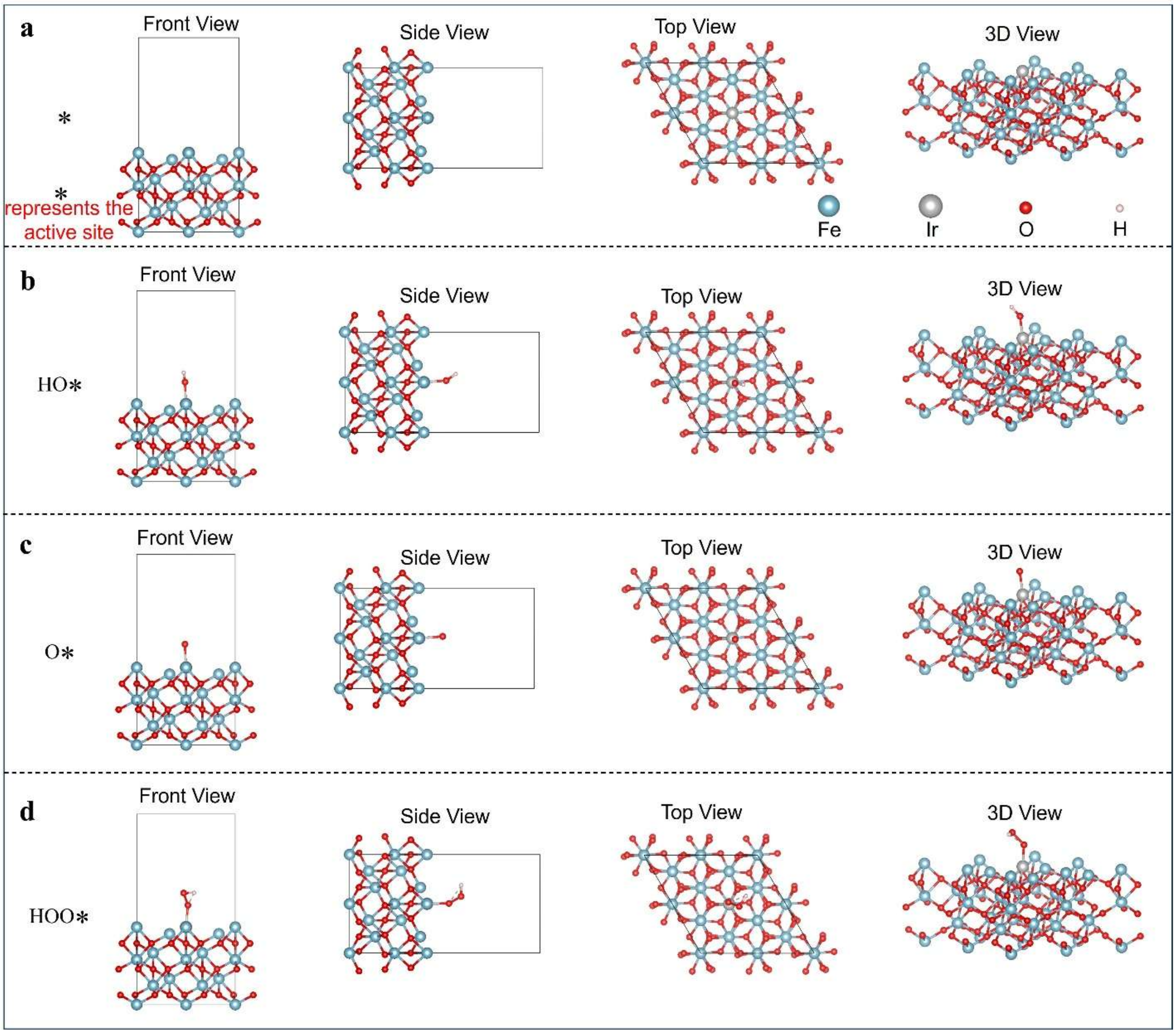

Fig. S38. Structural models for different states during oxygen evolution on the Ir single-atom catalyst based on the single-metal site mechanism. (**a**) Initial OER state with single Ir active sites, shown from four viewing angles. (**b**) OH adsorption on one Ir site, shown from four viewing angles. (**c**) O adsorption on one Ir sites, shown from four viewing angles. (**d**) OOH adsorption on the one Ir site, shown from four viewing angles.

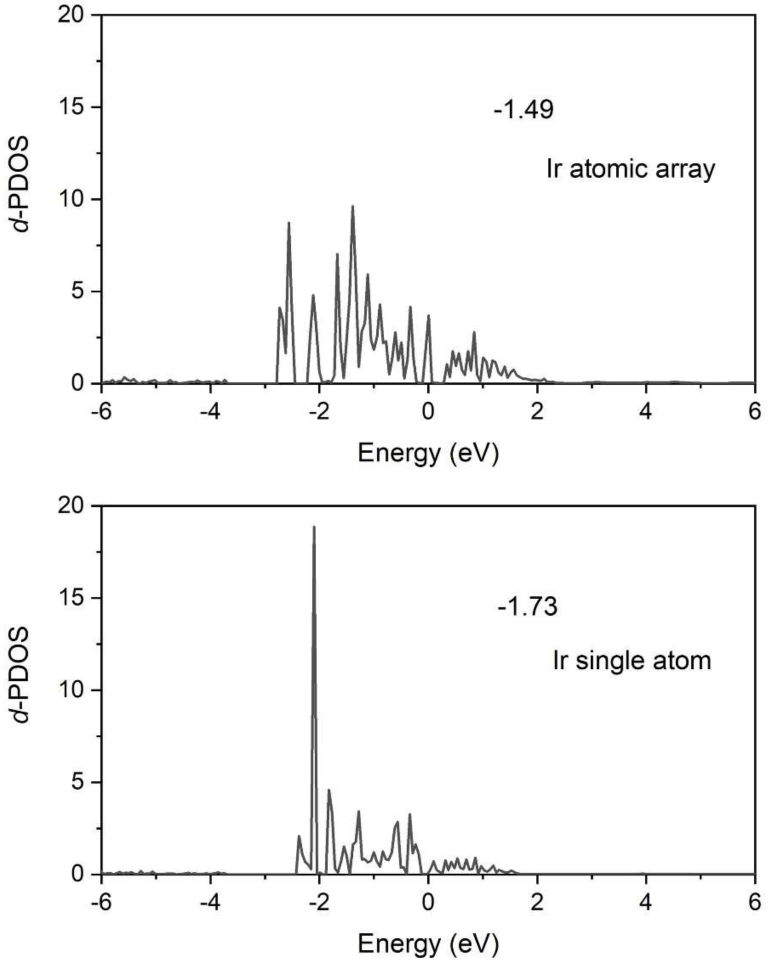

Fig. S39. The projected density of states on the d orbitals (d-PDOS) of Ir atoms in the Ir atomic patches and Ir single-atom model. The values of the d-band centers (-1.49 and -1.73) were obtained based on the results of PDOS.

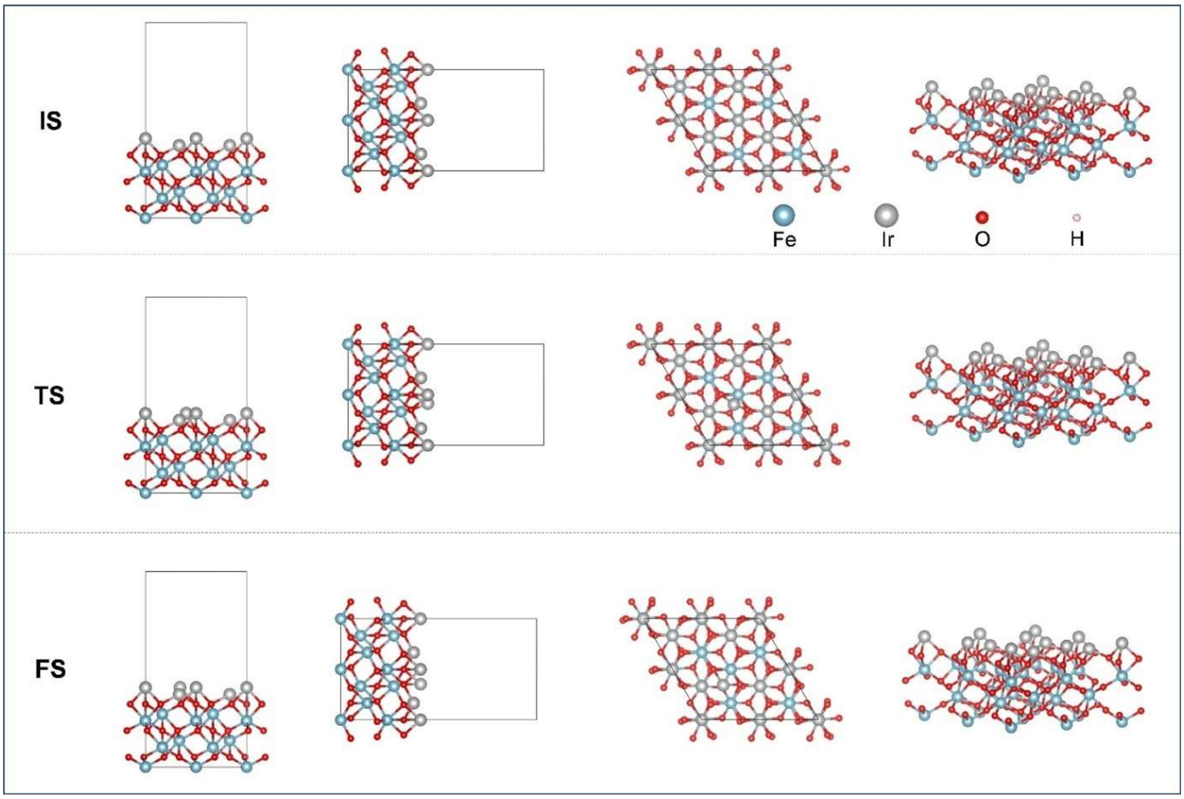

Fig. S40. Structural models of Ir atomic patches at different angles under different migration states of surface Ir atoms.

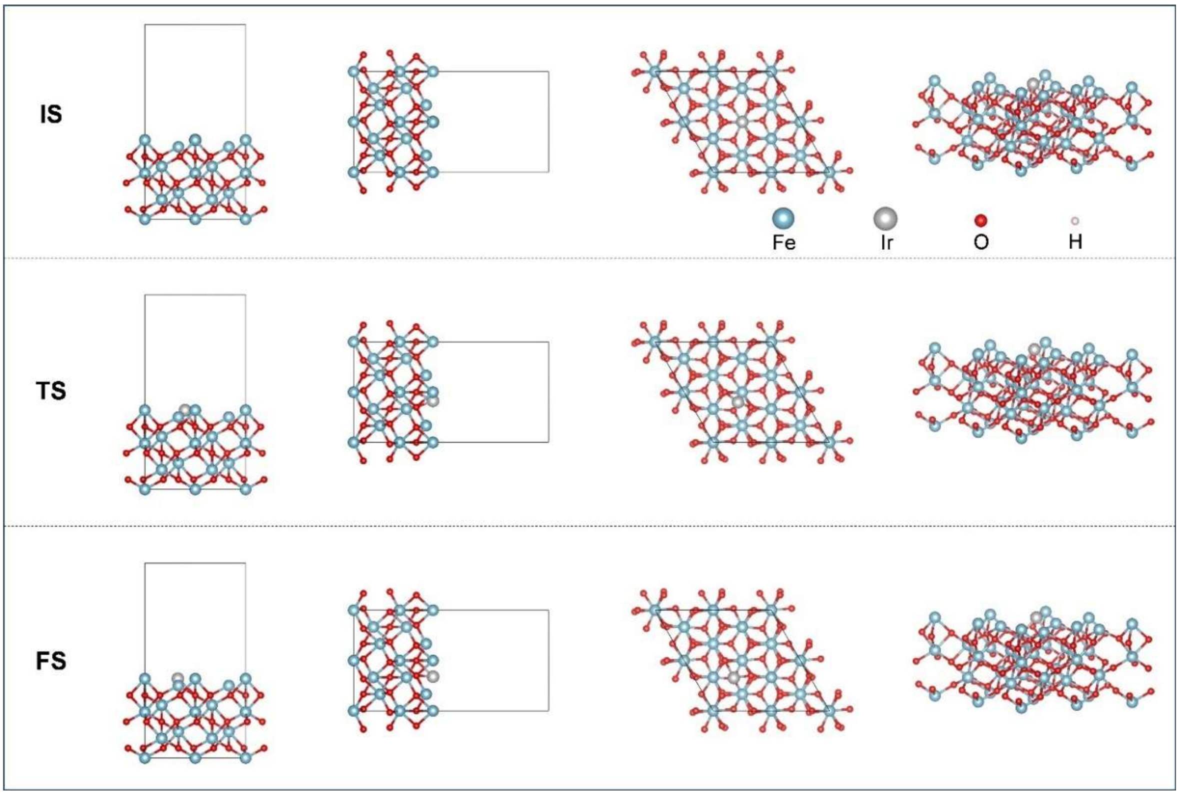

Fig. S41. Structural models of Ir single-atom catalyst at different angles under different migration states of surface Ir atom.

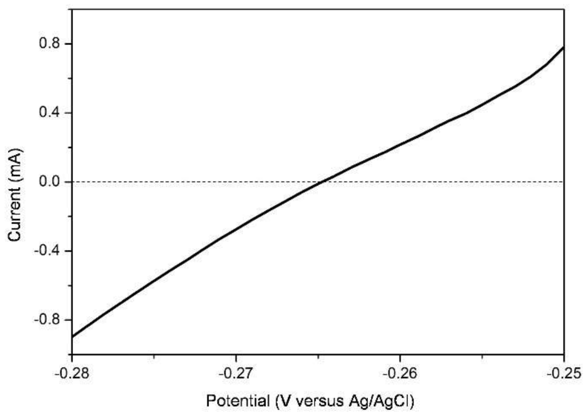

Fig. S42. Current-potential curve for the calibration of the Ag/AgCl with respect to the RHE in H_2-saturated 0.1 M $HClO_4$ solution using a Pt foil as the working electrode. The value of the potential at a current density of zero was regarded as the thermodynamic potential for the electrocatalytic hydrogen electrode reaction. Thus, the potential with respect to the RHE can be calculated as follows: *E*(RHE) = *E*(Ag/AgCl) + 0.265 V.

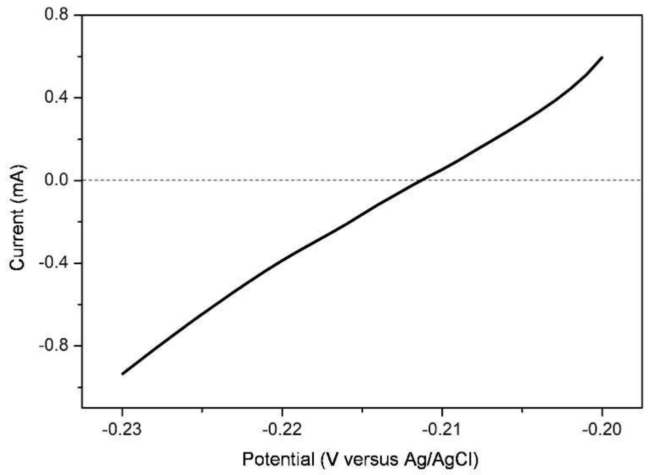

Fig. S43. Current-potential curve for the calibration of the Ag/AgCl with respect to the RHE in H_2-saturated 1 M $HClO_4$ solution using a Pt foil as the working electrode. The value of the potential at a current density of zero was regarded as the thermodynamic potential for the electrocatalytic hydrogen electrode reaction. Thus, the potential with respect to the RHE can be calculated as follows: E(RHE) = E(Ag/AgCl) + 0.212 V.

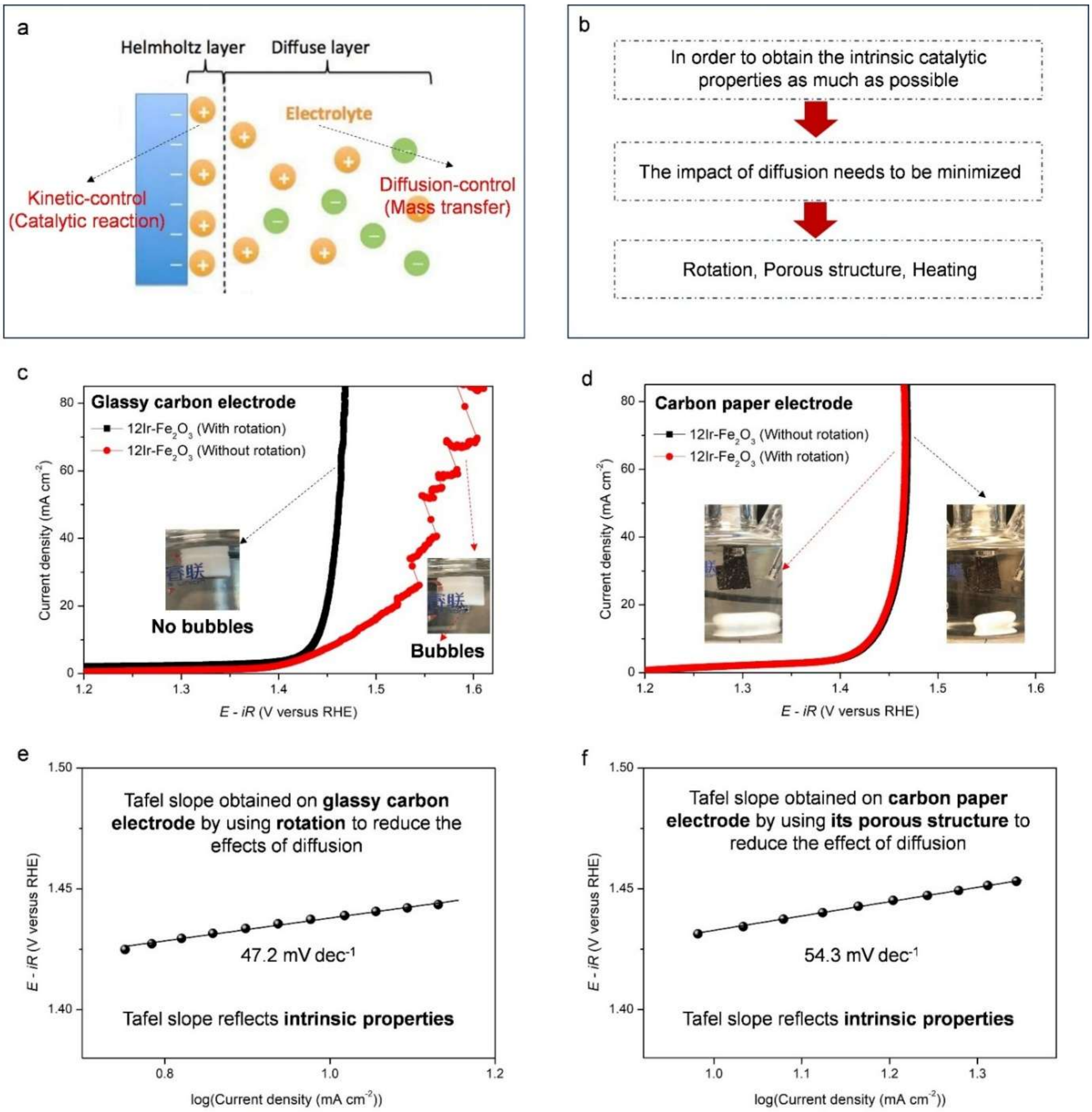

Fig. S44. (**a**) Schematic diagram of the electrochemical double layer model. (**b**) Schematic diagram of the relationship between rotation and diffusion in electrocatalysis. (**c**) OER polarization curves of 12Ir-Fe_2O_3 in rotating and non-rotating states on glassy carbon electrode. (**d**) OER polarization curves of 12Ir-Fe_2O_3 in rotating and non-rotating states on carbon paper electrode. (**e**) Tafel slope derived from **c** (with rotation). (**f**) Tafel slope derived from **d**.

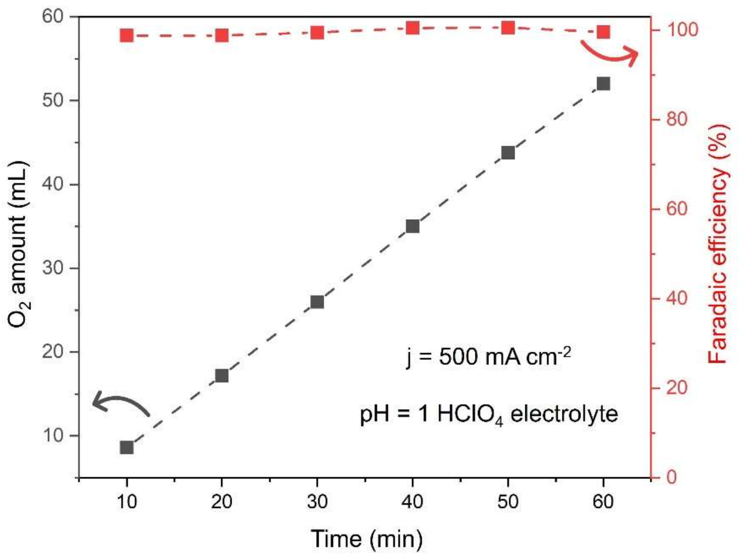

Fig. S45. Faradaic efficiency (red square) and corresponding oxygen evolution volume (black square) of 12Ir–Fe_2O_3 measured at a current density of 500 mA cm^{-2} in $HClO_4$ electrolyte.

Supplementary Tables

Table S1. EXAFS fitting parameters at the Ir L_3-edge for various samples (S_0^2=0.85).

Sample	Shell	*CN*[a]	*R*(Å)[b]	σ^2 (Å^2·10^{-3})[c]	ΔE_0 (eV)[d]	R factor (%)
2Ir-Fe_2O_3	Ir-O	5.8±0.56	2.00±0.05	0.007	8.47±1.05	0.60
	Ir-Fe	3.5±1.94	3.02±0.07	0.007		
6Ir-Fe_2O_3	Ir-O	5.8±0.65	2.01±0.01	0.005	9.58±1.14	0.77
	Ir-Fe	0.9±1.16	3.01±0.05	0.003		
	Ir-Ir	0.6±0.39	3.11±0.07	0.003		
12Ir-Fe_2O_3	Ir-O	5.9±0.66	2.01±0.01	0.005	9.88±1.08	0.73
	Ir-Fe	0.4±0.02	2.94±0.00	0.003		
	Ir-Ir	1.9±1.18	3.14±0.20	0.025		

[a]*CN*, coordination number; [b]*R*, distance between absorber and backscatter atoms; [c]σ^2, Debye-Waller factor to account for both thermal and structural disorders; [d]ΔE_0, inner potential correction; *R* factor indicates the goodness of the fit. Fitting range: 3.0 < k (Å^{-1}) < 11.0 and 1.0 < R (Å)< 3.0.

Table S2. Comparison of OER catalytic performance with previously reported noble-metal-based electrocatalysts in acidic media.

Catalyst	pH of electrolyte	Mass loading of precious metals ($\mu g\ cm^{-2}$)	Overpotential at 10 mA cm^{-2} (mV)	Stability at 10 mA cm^{-2} (hours)	Ref.
**12Ir-Fe_2O_3**	**0**	**11.7**	**198 (with iR correction)**	**1600**	**this work**
**12Ir-Fe_2O_3**	**1**	**11.7**	**216 (with iR correction)**	**1500**	**this work**
12Ru/MnO_2	1	22	161	200	33
$Ir_1/Cu_{0.3}Co_{2.7}O_4$	1	72	290	60	16
Ti-IrO_x/Ir	0	325	254	100	54
$Ta_{0.1}Tm_{0.1}Ir_{0.8}O_{2-\delta}$	0	19.3	198	500	12
Ni-RuO_2	1	225.6	214	205	14
IrO_2NR	0	191	205	138	10
Ir-MnO_2	0	51.3	218	650	27
Ir-$NiCo_2O_4$ NSs	0	10.4	240	70	53
Ir-Co_3O_4	0	18	236	30	52
$RuNi_2$@G-250	0	55.7	270	24	50
Amor. Ir NSs	1	204	255	8	49
$Y_2Ru_2O_{7-x}$	1	10.6	310	8	48
Ru_1-Pt_3Cu	1	16.3	220	28	51
IrO_2@Ir/TiN	0	223.6	265	6	47
IrO_2/GCN	0	70.2	276	4	46
AA-IrO_2	0	20	370	1	41
Ba_2YIrO_6	1	4.4	318	1	43

$Nd_2Ir_2O_7$	1	27.8	325	2.8	44
$Pr_2Ir_2O_7$	1	28.1	300	2.8	44
IrNi NCs	1	12.5	280	2	45
$SrCo_{0.9}Ir_{0.1}O_{3-x}$	1	23	350	3	42
